# Strict Recursive Closed-Loop Evaluation of Greenhouse Air Temperature Forecasting with Generated Exogenous Variables and Exogenous-Error Propagation Analysis

**DOI:** 10.3390/s26154717

**Published:** 2026-07-24

**Authors:** Aiguang Zhang, Shuo Zhang, Jingyu Bian, Jianfei Xing, Wentao Li, Hong Gu, Xufeng Wang

**Affiliations:** 1 College of Mechanical and Electrical Engineering, Tarim University, Alar 843300, China; 10757242266@stumail.taru.edu.cn (A.Z.); 10757241148@stumail.taru.edu.cn (S.Z.); 10757242258@stumail.taru.edu.cn (J.B.); 10757242264@taru.edu.cn (J.X.); 10757242257@taru.edu.cn (W.L.); 2 Xinjiang Production and Construction Corps (XPCC), Key Laboratory of Utilization and Equipment of Special Agricultural and Forestry Products in Southern Xinjiang, Alar 843300, China; 3 Modern Agricultural Engineering Key Laboratory at Universities of Education Department of Xinjiang Uygur Autonomous Region, Alar 843300, China

**Keywords:** environmental sensors, greenhouse air temperature forecasting, recursive closed-loop evaluation, exogenous-variable generation, exogenous-error propagation, multi-horizon forecasting

## Abstract

Accurate multi-horizon forecasting of greenhouse air temperature is essential for sensor-based environmental monitoring and control-oriented decision support. However, conventional evaluation methods often rely on open-loop or semi-closed-loop settings, where future observations may be unavailable during practical deployment. This study established a strict recursive closed-loop forecasting protocol and an exogenous-error propagation analysis framework using multi-source environmental sensing data. A Liquid Neural Network (LNN) was developed as a continuous-time forecasting model and compared with long short-term memory (LSTM), gated recurrent unit (GRU), and extreme gradient boosting (XGBoost) under consistent data conditions. During inference, future greenhouse air temperature and exogenous variables were not observed but recursively generated through predicted-temperature feedback and exogenous-variable forecasting. Across 10 repeated runs, the LNN achieved the lowest one-step prediction error, with a mean absolute error of 0.4771 °C and root mean square error of 0.5996 °C. However, under strict recursive closed-loop forecasting, LSTM showed the lowest errors at 6 h, 24 h, and 48 h horizons, indicating stronger long-horizon stability. Ablation experiments demonstrated that using observed future exogenous variables underestimated forecasting errors, while exogenous-error analysis identified outdoor temperature and solar radiation as the dominant sources of propagated uncertainty. These results highlight the importance of evaluating greenhouse air temperature forecasting under realistic recursive conditions by considering both model stability and future input uncertainty.

## 1. Introduction

Greenhouse air temperature is a key state variable in environmental regulation for protected cultivation. It directly affects crop photosynthesis, transpiration, nutrient uptake, flower and fruit development, and disease risk [1]. With advances in Internet of Things (IoT) sensors, wireless communication, and environmental monitoring platforms, indoor and outdoor air temperature and humidity, solar radiation, wind speed, soil moisture, and CO_2_ concentration can be continuously collected in greenhouses, forming high-temporal-resolution multi-source environmental sensing data. These data provide an important basis for greenhouse air temperature forecasting, environmental state monitoring, and intelligent regulation. Unlike open-field environments, greenhouse air temperature is not simply determined by external meteorological conditions, but is jointly affected by solar radiation, heat transfer through covering materials, ventilation heat exchange, heat storage and release by soil and walls, crop canopy transpiration, and production management practices [2]. Therefore, greenhouse air temperature forecasting models should not only characterize the thermal state of the greenhouse using multi-source sensing data, but also provide continuous temperature trajectories over future decision windows ranging from several hours to several days [3,4]. Such forecasts are practically valuable because greenhouse control actions, including ventilation, shading, auxiliary heating, and other environmental regulation measures, often require lead time and are affected by facility thermal inertia. Reliable multi-horizon temperature trajectories can therefore support early warning of high- or low-temperature risks, anticipatory environmental regulation, and model predictive control, rather than relying only on delayed threshold-based responses.

More broadly, data-driven modelling has become an important methodological basis for digital agriculture and precision-farming control. Statistical learning, data mining, predictive modelling, and machine-learning approaches can transform historical sensing data into predictions and decision-support information for optimized agricultural management [5]. Recent reviews have further emphasized that the integration of machine learning, optimization techniques, cyber–physical systems, information and communication technologies, and control theory is reshaping agricultural system modelling by improving decision-making, resource-use efficiency, and sustainability [6]. These developments provide a general background for using multi-source sensing data and recursive forecasting models to support greenhouse environmental prediction and control-oriented decision-making.

Data-driven methods have been widely used for greenhouse microclimate forecasting. Early studies demonstrated that neural networks can characterize nonlinear relationships among greenhouse environmental variables and can be used for greenhouse air temperature prediction [7,8]. In recent years, with the accumulation of greenhouse IoT monitoring data, machine-learning and deep-learning models have been applied to multi-parameter forecasting tasks involving greenhouse temperature, humidity, CO_2_ concentration, solar radiation, and soil environmental variables [9,10,11]. Francik and Kurpaska constructed an artificial neural network (ANN) model based on facility environmental monitoring data to forecast air temperature changes inside a heated foil tunnel, showing that neural-network models based on indoor and outdoor environmental sensing variables can support facility microclimate prediction and environmental management [12]. Codeluppi et al. further investigated greenhouse indoor air temperature forecasting from the perspective of edge deployment by combining IoT monitoring data with embedded artificial intelligence models [13]. These studies indicate that sensor-data-driven greenhouse forecasting has extended from environmental monitoring itself to predictive modelling, edge deployment, and control-oriented decision support. For multi-horizon forecasting, attention-based LSTM models, Transformer/recurrent neural network (RNN) comparison studies, and gated recurrent unit (GRU) models based on external weather data have shown that deep sequence models can capture historical dependence and nonlinear variation trends in greenhouse environmental variables [14,15,16]. However, most existing studies still focus on offline prediction accuracy, while recursive stability under online deployment, the unavailability of future sensor observations, and long-horizon error accumulation remain insufficiently investigated.

Existing greenhouse air temperature forecasting studies still show a mismatch between evaluation protocols and practical application scenarios. Many studies adopt one-step prediction or open-loop multi-step prediction, in which observed sequences are used to update the input window when forecasting future states, or future exogenous variables such as outdoor meteorological variables, solar radiation, and humidity are assumed to be directly available. In practical online applications, however, the model can access only historical sensor observations collected before the forecast origin. As rolling forecasting proceeds, future greenhouse air temperature cannot be provided in advance by sensors and must be obtained through model prediction and feedback, while future driving variables must be supplied by weather forecasts, exogenous-variable generators, or other estimation methods. For related sensor-estimation tasks, Guesbaya et al. constructed an LSTM-RNN-based soft sensor for greenhouse vent opening estimation using indoor and outdoor greenhouse climate sensing variables, indicating that sensor-data-based models can be used not only for environmental variable prediction but also for indirect estimation of unobserved states [17]. Soft-sensor studies have also shown that the integration of hardware sensing data and computational models can estimate variables that are difficult to directly measure or obtain in real time, thereby improving biosystem monitoring and control capability [18]. Therefore, in multi-horizon greenhouse air temperature forecasting, using observed future temperature or observed future exogenous sensor variables during evaluation may underestimate long-horizon prediction errors and overestimate model deployability. Multi-step time-series forecasting studies have shown that direct, recursive, and multi-output strategies have different error propagation mechanisms [19], while sequence-generation studies have indicated that relying on observed inputs during training but model-generated outputs during inference can induce training–inference mismatch and error accumulation [20]. Thus, online-deployment-oriented evaluation of greenhouse air temperature forecasting should simultaneously consider predicted-temperature feedback and uncertainty in future exogenous sensor variables [21,22,23].

From the perspective of greenhouse thermal dynamics, air temperature exhibits continuous evolution, multiple time scales, and strong exogenous forcing. Solar radiation, ventilation disturbances, and changes in outdoor temperature and humidity can induce short-term temperature fluctuations, whereas walls, soil, covering materials, and crop canopies introduce thermal inertia and delayed responses. LSTM, GRU, and extreme gradient boosting (XGBoost) have been widely used for time-series modelling and nonlinear prediction tasks [24,25,26], but their error accumulation characteristics under strict recursive closed-loop conditions still require further comparison. In recent years, continuous-time neural networks, including neural ordinary differential equations, latent ODEs, liquid time-constant networks, and neural controlled differential equations, have provided new methodological foundations for modelling dynamic state evolution [27,28,29,30]. Liquid Neural Networks (LNNs), as a class of continuous-time recurrent neural models, describe system dynamic responses through learnable time constants and continuous state updates. These properties make LNNs a suitable continuous-time candidate model for examining greenhouse air temperature forecasting under recursive conditions, because greenhouse temperature sequences are characterized by coexisting fast and slow processes, thermal inertia, and strong exogenous forcing. However, whether such short-term dynamic fitting ability can be maintained during long-horizon recursive forecasting remains uncertain when predicted-temperature feedback, generated exogenous variables, and error accumulation are simultaneously involved.

To address these issues, this study established a strict recursive closed-loop forecasting protocol and an exogenous-error propagation analysis framework for greenhouse air temperature prediction using multi-source environmental sensing data. Within this framework, an LNN model was constructed as a continuous-time forecasting model to evaluate the feasibility of recursive closed-loop prediction, and LSTM, GRU, and XGBoost were introduced as comparison models under the same information conditions. During inference, the model did not use observed future greenhouse air temperature or observed future exogenous variables. Instead, predicted temperatures were progressively fed back into the input window, and future exogenous variables were recursively generated by an exogenous-variable generator, thereby simulating an autonomous forecasting process closer to practical online deployment. Under unified data partitioning, input variables, historical window length, and evaluation metrics, LNN, LSTM, GRU, and XGBoost were evaluated in one-step prediction and in strict recursive closed-loop forecasting over 6 h, 24 h, and 48 h horizons. In addition, exogenous-variable generation errors and their correlations with greenhouse air temperature prediction errors were analysed, and protocol ablation experiments were conducted to examine how different future exogenous-variable acquisition methods affect multi-horizon forecasting performance. The aim of this study was therefore to provide a deployment-oriented recursive evaluation protocol for greenhouse air temperature forecasting and to reveal how errors from generated exogenous variables propagate into long-horizon temperature prediction when future sensor observations are unavailable.

## 2. Materials and Methods

### 2.1. Data Source

The experiment was conducted in a solar greenhouse used for tomato production from 1 January 2024 to 30 December 2024. The sensor sampling interval was 30 min, and 17,520 raw time-series records were obtained. Each record represented the sensor reading stored at the corresponding 30 min timestamp; wind speed and photosynthetically active radiation (PAR) were therefore used as instantaneous recorded values rather than 30 min averaged values. The greenhouse was 100 m long in the east–west direction, 12 m wide in the north–south direction, and 8 m high at the ridge. It was equipped with common production management facilities, including a roller-shutter system and water-fertilizer irrigation equipment.

**Figure 1:**
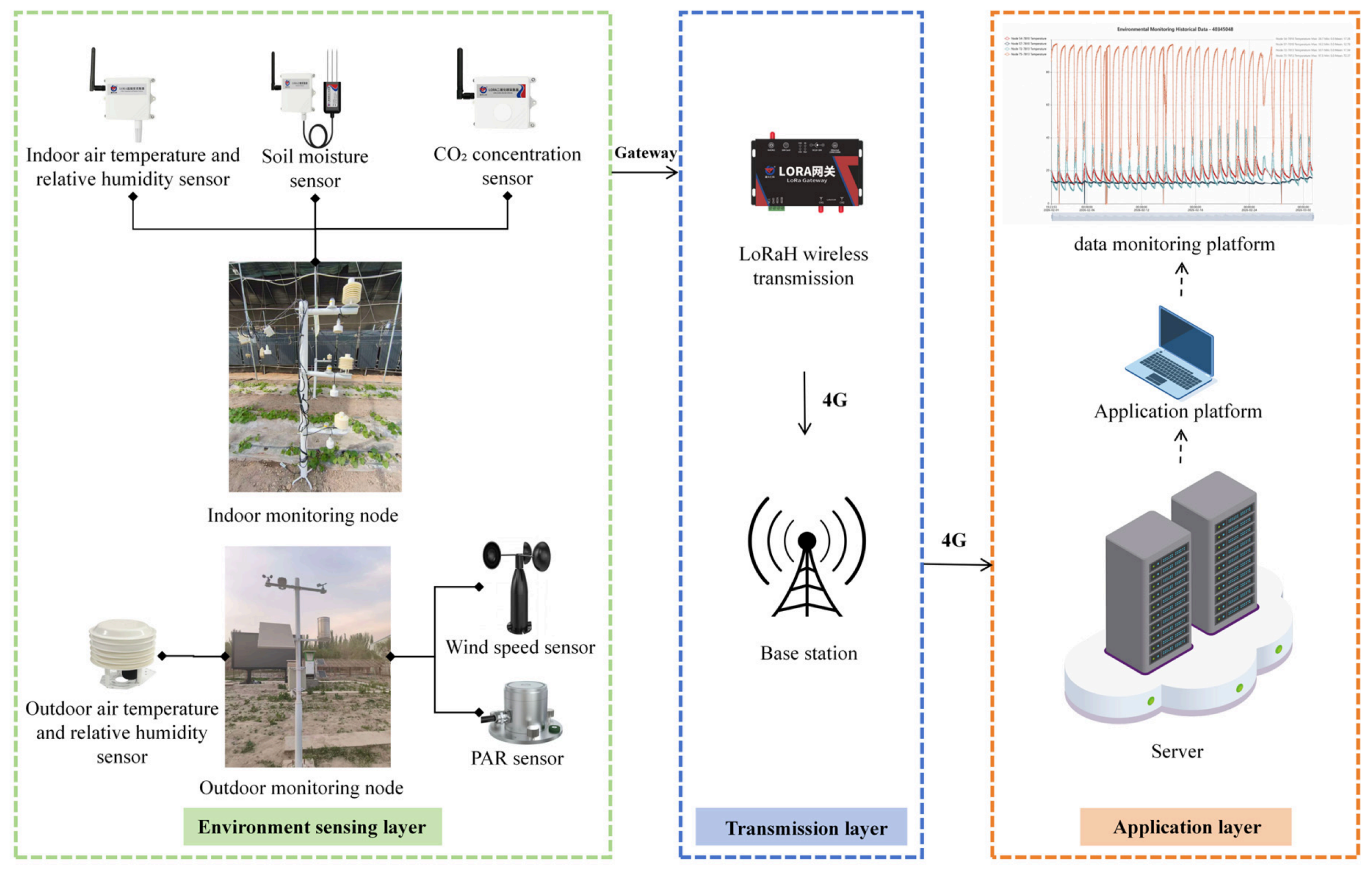
Greenhouse environmental monitoring and data acquisition system.

To construct a multi-source environmental sensing dataset for greenhouse air temperature forecasting, environmental data acquisition nodes were installed inside the greenhouse at 20, 40, 60, and 80 m along the east–west direction. The sensors were mounted at a height of 1.5 m to continuously collect indoor air temperature, indoor relative humidity, and CO_2_ concentration. Soil moisture sensors were installed inside the greenhouse at a depth of 20 cm to characterize the root-zone water status. Outdoor environmental data were collected using a small weather station, including outdoor air temperature, outdoor relative humidity, photosynthetically active radiation (PAR), and wind speed. In this study, PAR was used to represent the solar-radiation variable in the forecasting model. All environmental sensors used in this study were manufactured by Shandong JianDa RenKe Measurement and Control Technology Co., Ltd. (Weifang, China). Together, the indoor and outdoor sensors constituted a multi-source greenhouse environmental monitoring system, providing historical state variables and exogenous driving variables for greenhouse air temperature forecasting. The main technical specifications of the sensors are listed in Table 1. The collected data were transmitted from the sensor nodes to an Internet of Things gateway, uploaded to a server via a 4G network, and stored in an environmental monitoring database. The sensor monitoring data were then processed through missing-value imputation, outlier screening, feature construction, and standardization before being used for one-step prediction, strict recursive closed-loop forecasting, and exogenous-error propagation analysis. The greenhouse environmental data acquisition process is shown in Figure 1.

### 2.2. Data Preprocessing

This study used time-series data continuously collected by multi-source greenhouse environmental sensors. Previous greenhouse microclimate forecasting studies have commonly incorporated indoor and outdoor environmental variables, such as air temperature, relative humidity, solar radiation, wind speed, CO_2_ concentration, and soil-related variables, as model inputs to characterize the coupled effects of internal environmental states and external meteorological forcing on greenhouse temperature and humidity dynamics [31,32]. In this study, the collected variables included indoor air temperature, indoor relative humidity, outdoor air temperature, outdoor relative humidity, PAR, wind speed, soil moisture, CO_2_ concentration, and vapor pressure deficit (VPD). PAR was used to represent the solar-radiation input variable, and VPD was calculated as a derived variable from air temperature and relative humidity. The greenhouse air temperature at the next sampling time was used as the prediction target. Historical indoor air temperature and its derived features, indoor relative humidity, outdoor meteorological variables, PAR, wind speed, soil moisture, CO_2_ concentration, and VPD were jointly used as model inputs to characterize the historical thermal state, external meteorological boundary conditions, internal hydrothermal conditions, and sensor observation conditions of the greenhouse.

#### 2.2.1. Missing Value Processing

During long-term continuous data acquisition, a small number of missing values may occur because of sensor communication delays, network transmission failures, or short-term device downtime. The continuity of timestamps and the completeness of variables were first checked. Short missing segments were filled using linear interpolation. For a small number of missing points at the boundaries of the sequence, where direct interpolation was not possible, the nearest valid value was used for filling. Data segments with long continuous missing periods that could not be reliably reconstructed were not used to construct valid prediction samples. Linear interpolation was calculated as follows:

(1) $$x_{t}\;=\;x_{t_{1}}+\frac{t-t_{1}}{t_{2}-t_{1}}(x_{t_{2}}-x_{t_{1}})$$

where $x_{t}$ denotes the interpolated value at the missing time t, and $x_{t1}$ and $x_{t2}$ denote the adjacent valid observations before and after the missing point, respectively.

#### 2.2.2. Outlier Processing

Outliers were identified based on the physical range of each variable and the continuity between adjacent time points. Observations outside a reasonable physical range were marked as outliers. These outliers were first set as missing values and then corrected using the same interpolation or nearest-valid-value filling method as that used for missing values. This procedure can be expressed as follows:

(2) $$x_{t}^{*}\;=\;\left\{\begin{array}{c} NaN,\;x_{t}\;<\;x_{\min}\;or\;x_{t}\;>\;x_{\max}\\ x_{t},{\;x}_{\min}\;\leq {\;x}_{t}\;\leq {\;x}_{\max} \end{array}\right.$$

where $x_{t}$ is the original observation, $x_{t}^{*}$ is the screened value, and $x_{min}$ and $x_{max}$ are the lower and upper physical bounds of the corresponding variable, respectively.

#### 2.2.3. Input Feature Construction

After data cleaning, model input features were constructed from raw environmental variables, time-periodic features, lagged features, and derived features. Raw environmental variables described the internal greenhouse state and external environmental forcing. Time-periodic features included sine and cosine encodings of hour of day and day of year, representing diurnal and annual periodicity. Lagged features were used to describe historical dependence and thermal inertia in the greenhouse thermal environment. Derived features included the indoor–outdoor temperature difference, PAR-related interaction terms, lagged PAR terms, lagged outdoor temperature terms, temperature slopes, and other variables. These features were designed to improve the representation of temperature trends and delayed thermal responses in the greenhouse.

#### 2.2.4. Data Standardization

Because the variables differed substantially in units and numerical ranges, both the input variables and the prediction target were standardized. The dataset was divided into fixed training, validation, and test sets at a ratio of 70:15:15, and the same split was used for all models and repeated runs. The standardization parameters were calculated only from the training set and then applied to the validation and test sets to avoid data leakage. Standardization was performed as follows:

(3) $$x^{\mathrm{std}}\;=\;\frac{x-\mu_{x}}{\sigma_{x}}$$

where x is the original variable, $x^{std}$ is the standardized variable, and $\mu_{x}$ and $\sigma_{x}$ are the mean and standard deviation of the corresponding variable calculated from the training set, respectively.

### 2.3. Greenhouse Air Temperature Forecasting Model

#### 2.3.1. Liquid Neural Network

Within the proposed strict recursive closed-loop forecasting framework, a Liquid Neural Network (LNN) was constructed as a recurrent forecasting model based on continuous-time dynamics. It was used to examine whether continuous state updates and learnable time constants can support recursive greenhouse air temperature prediction under multi-source environmental forcing. Unlike conventional discrete-time recurrent neural networks, the LNN updates hidden states through learnable time constants and nonlinear state equations. This structure provides a theoretically relevant modelling strategy for greenhouse thermal processes affected by solar radiation, outdoor meteorological conditions, soil moisture, and facility thermal inertia, while its long-horizon recursive stability needs to be evaluated through the proposed closed-loop protocol.

**Figure 2:**
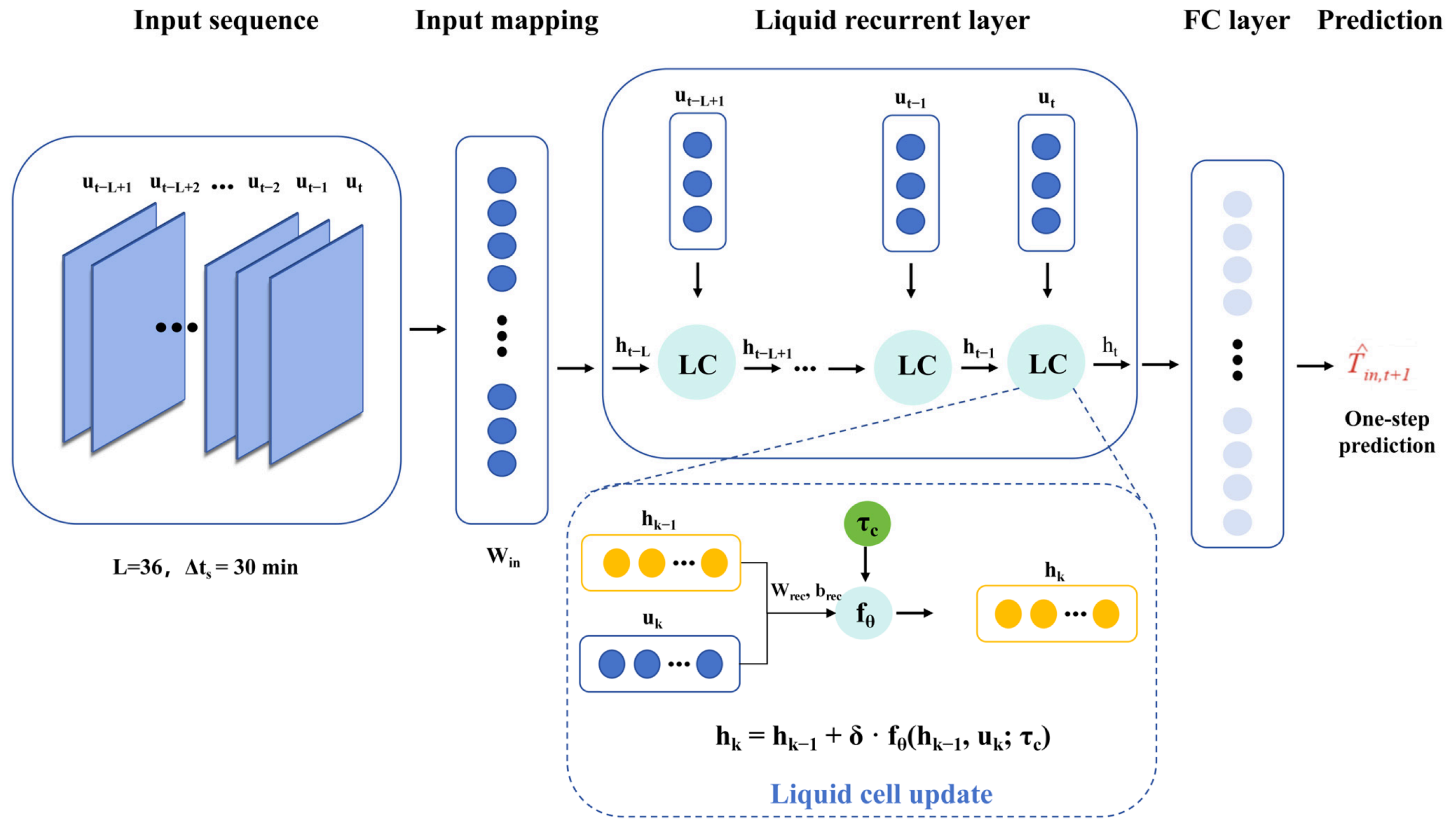
Overall architecture of the proposed LNN for greenhouse air temperature forecasting: The model uses a historical multivariate environmental sequence with L = 36 and Δt_s_ = 30 min as input. The input sequence is mapped into the liquid dynamics layer through the input weight matrix *W*_in_. Liquid cells (LCs) update their hidden states using the current input, the previous hidden state, and learnable time constants. After the complete historical window is processed, the final hidden state is passed to the output prediction layer to predict greenhouse air temperature at the next sampling time.

As shown in Figure 2, the proposed LNN model consists of four components: an input feature layer, a mapping layer, a liquid dynamics layer, and an output prediction layer. The model takes a historical multivariate environmental sequence with a length of L = 36 as input. Because the sampling interval is Δt_s_ = 30 min, each input window covers the environmental state over the past 18 h. The prediction target is the greenhouse air temperature at the next sampling time.

(1) Input feature layer

The input feature layer was used to construct the historical state sequence of the greenhouse environment. At the k-th sampling point, the model input feature vector *u_k_* included raw environmental variables such as outdoor temperature, outdoor relative humidity, solar radiation, wind speed, soil moisture, CO_2_ concentration, VPD, and indoor relative humidity. It also included derived features constructed from historical indoor and outdoor temperatures, including the temperature difference, solar-radiation interaction terms, lagged radiation terms, lagged outdoor temperature terms, temperature slopes, and time-periodic encodings. These features jointly represented the external meteorological forcing, internal hydrothermal state, historical lag effects, and diurnal periodicity affecting greenhouse air temperature.

(2) Input mapping

The mapping layer was used to project the multivariate environmental input into the liquid recurrent dynamics layer. In this study, the input features were not transformed by an independent feed-forward hidden layer. Instead, they entered the liquid state equation directly through the input weight matrix *W*_in_, serving as external driving terms for hidden-state updates. The continuous-time hidden-state vector is defined in Equation (4), and the state evolution equation is given in Equation (5):

(4) $${h(t)=[h_{1}(t),\;h_{2}(t),\;\ldots ,\;h_{N}(t)]}^{t}$$

(5) $$\frac{dh(t)}{dt}=\frac{-h(t)+W_{\mathrm{in}}u(t)+W_{\mathrm{rec}}h(t)+b_{\mathrm{rec}}+\alpha ⨀{h(t)}^{2}-\beta ⨀{h(t)}^{3}}{\max(\tau_{c},\;\tau_{\min})}$$

(3) Liquid recurrent layer

The liquid dynamics layer is the core of the LNN and consists of 64 liquid neurons, corresponding to the hidden-state dimension selected in this study. Each liquid neuron corresponds to one component of the hidden-state vector, and all neurons are connected through the recurrent weight matrix $W_{\mathrm{rec}}$. At the k-th time step, the liquid cell receives the current input feature vector $u_{k}$ and the hidden state $h_{k}$, and then updates the next hidden state $h_{k+1}$ using the learnable time constant $\tau_{c}$, recurrent weight matrix $W_{\mathrm{rec}}$, state-update bias $b_{\mathrm{rec}}$, and nonlinear polynomial terms. The discretized update form of the continuous dynamics is shown in Equation (6). This structure enables the model to progressively accumulate dynamic information within the historical input window and compress the environmental changes over the past 18 h into the hidden-state representation at the end of the window.

(4) Output connected layer

The output prediction layer maps the hidden state obtained at the end of the liquid dynamics layer to the predicted greenhouse air temperature at the next sampling time. The model first obtains the prediction output in the standardized space through a linear readout structure and constrains the output range using a clipping function, as shown in Equation (7). The standardized output is then linearly calibrated using the validation set and inverse-standardized to the original physical scale, yielding the final greenhouse air temperature prediction ${\hat{T}}_{\mathrm{in},k+1}$. During strict recursive closed-loop forecasting, this predicted temperature is fed back into the simulated input sequence and combined with future exogenous variables recursively generated by the exogenous-variable generator to construct the next-step input, thereby forming a “prediction–feedback–prediction” recursive process.

During strict recursive closed-loop forecasting, after completing one-step prediction, the model feeds the predicted greenhouse air temperature ${\hat{T}}_{\mathrm{in},k+1}$ back into the simulated sequence. This predicted temperature is then combined with the future exogenous variables generated by the exogenous-variable generator to construct the next-step input. The closed-loop input reconstruction process is shown in Equation (8). Through this process, the model does not read observed future greenhouse air temperature or observed future exogenous variables during inference. Instead, it relies on its own predicted temperature and generated exogenous driving variables to perform continuous rolling forecasting.

The remaining equations of the LNN are as follows:

(6) $$h_{k+1}\;=\;\mathrm{clip}(h_{k}+\delta \frac{-h_{k}\;+\;W_{\mathrm{in}}u_{k}\;+\;W_{\mathrm{rec}}h_{k}\;+\;b_{\mathrm{rec}}\;+\;\alpha ⨀{h_{k}}^{2}-\beta ⨀{h_{k}}^{3}}{\max(\tau_{c},{\;\tau }_{\min})},-h_{\max},\;h_{\max})\;$$

(7) $${{\hat{y}}^{n}}_{k+1\;}=\mathrm{clip}(W_{\mathrm{out}}h_{k+1}+b_{\mathrm{out}},-y_{\max},\;y_{\max})$$

(8) $$u_{k+1}=F[{\hat{T}}_{\mathrm{in},\;k+1},\;u_{k+1}^{\mathrm{exo}},{\;\phi }_{k+1}]$$

where h(t) is the continuous-time hidden-state vector; $h_{i}(t)$ is the state of the i-th neuron; *N* is the hidden-state dimension; *t* is the continuous-time variable; u(t), $W_{in}$, $W_{\mathrm{rec}}$, and $b_{\mathrm{rec}}$ denote the input feature vector, input-state weight matrix, recurrent weight matrix, and state-update bias vector, respectively; α and β are polynomial nonlinear coefficients; $\tau_{c}$ is the time-constant vector; $\tau_{\min}$ is the lower bound of the time constant, set to 0.1 in this study; $\max(\tau_{c},\;\tau_{\min})$ denotes an element-wise lower-bound constraint; ⨀ denotes element-wise multiplication; *h*^2^ and *h*^3^ denote element-wise powers; *k* is the discrete sampling index; *δ* is the internal state-update step size of the LNN; *h_k_* and *u_k_* are the hidden state and input feature at the k-th sampling point, respectively; $\mathrm{clip}(\cdot ,-h_{\max},h_{\max})$ denotes an element-wise clipping function that constrains the hidden state within $\left[-h_{\max},h_{\max}\right]$, where $h_{\max}$ is the clipping threshold; ${{\hat{y}}^{n}}_{k\;+\;1\;}$ is the model output in the standardized space; $W_{\mathrm{out}}$ and $b_{\mathrm{out}}$ are the output-layer weight and bias parameters; ${\hat{T}}_{in,k+1}$ is the calibrated and inverse-standardized greenhouse air temperature prediction at the next sampling time; $u_{k+1}^{exo}$ is the future environmental driving vector generated by the exogenous-variable generator; $\phi_{k+1}$ contains the time-periodic encodings and related derived features; and F denotes the input feature reconstruction function in closed-loop forecasting.

Through the above structure, the LNN progressively updates its hidden state within the historical window and outputs the greenhouse air temperature at the next sampling time from the hidden state at the end of the window. During strict recursive closed-loop rolling inference, the model does not read observed future greenhouse air temperature. Instead, it feeds its own prediction back into the input sequence, while future exogenous variables are provided by the exogenous-variable generator. Therefore, the model can perform not only single-step temperature prediction but also strict recursive closed-loop rolling forecasting, which is used to evaluate the multi-horizon recursive performance of greenhouse air temperature prediction under practical online deployment conditions.

#### 2.3.2. Model and Training Parameter Settings

To evaluate the forecasting performance of the LNN, three comparison models were selected: LSTM, GRU, and XGBoost. LSTM states go through input, forget, and output gates, making it suitable for modelling long-term temporal dependence. GRU simplifies the recurrent structure using update and reset gates, allowing sequence dynamics to be modeled with fewer parameters. XGBoost was used as a non-sequential tree-based model to provide a strong nonlinear machine-learning baseline.

All models used the same data partitioning, input variables, historical window length, and evaluation metrics. The inputs of LNN, LSTM, and GRU were three-dimensional temporal tensors. For XGBoost, the multivariate sequence within each historical window was flattened in chronological order into a two-dimensional static feature vector. This setting ensured that all models were compared under the same information conditions and avoided evaluation bias caused by differences in input information.

The hidden-state dimension of the LNN was set to 64. This configuration was selected by considering the recursive forecasting performance, model complexity, training stability, and overfitting risk under different key hyperparameter settings based on a one-factor sensitivity analysis, as detailed in Table A1. The model was trained using the AdamW optimizer, and weight decay was introduced to reduce the risk of overfitting. The complete model architectures and training settings, including the optimizer, learning rate, batch size, number of epochs, loss function, regularization, learning-rate schedule, and model-selection criteria, are summarized in Table A2.

To ensure a consistent and fair comparison among the four forecasting models, model-specific one-factor-at-a-time hyperparameter sensitivity analyses were conducted using the validation set. Four representative hyperparameters were examined for each model, while the remaining settings were fixed at their reference values. Each candidate setting was evaluated using three fixed random seeds, and the MAE and RMSE at the 6 h, 24 h, and 48 h recursive forecasting horizons were considered jointly. When the overall recursive error of two candidate settings differed by less than 1%, they were regarded as practically comparable, and the configuration with lower run-to-run variability, lower model complexity, or stronger regularization was retained. The test set was not used during hyperparameter selection. The detailed sensitivity results are provided in Supplementary Table S4. All model development, training, and data analysis procedures were performed using MATLAB R2024a.

#### 2.3.3. Model Evaluation

To verify the forecasting performance of the LNN under the strict recursive closed-loop setting, root mean square error (RMSE) and mean absolute error (MAE) were used as the main evaluation metrics. All metrics were calculated after calibration and inverse standardization in the original physical scale. RMSE and MAE were calculated as follows:

(9) $$\begin{array}{c} RMSE\;=\sqrt{\frac{1}{N}\sum_{i=1}^{N}{\left({\hat{z}}_{in,i}-z_{in,i}\right)}^{2}} \end{array}$$

(10) $$\begin{array}{c} MAE=\frac{1}{N}\sum_{i=1}^{N}\left|{\hat{z}}_{in,i}-z_{in,i}\right| \end{array}$$

where *N* is the number of samples, and ${\hat{z}}_{in,i}$ and $z_{in,i}$ are the predicted and observed values of the i-th sample, respectively.

For one-step prediction, all next-step prediction samples in the test set were evaluated. For strict recursive closed-loop multi-horizon forecasting, MAE and RMSE were calculated over all rolling prediction steps within the 6 h, 24 h, and 48 h forecasting windows. These metrics were used to evaluate error accumulation and recursive stability under different forecasting horizons.

To account for training randomness, the complete training and evaluation procedure was repeated using 10 fixed random seeds (42, 123, 456, 789, 1024, 2024, 2025, 2026, 2048, and 3407), and model performance was reported as mean ± standard deviation. Overall differences among the models were evaluated using the Friedman test. When the overall test was significant, pairwise comparisons were conducted using two-sided Wilcoxon signed-rank tests with Holm correction, with statistical significance determined at *p* < 0.05. The complete run-level results and pairwise statistical comparisons are provided in Supplementary Materials.

### 2.4. Strict Recursive Closed-Loop Forecasting and Exogenous-Variable Generation

#### 2.4.1. Strict Recursive Closed-Loop Forecasting Strategy

To evaluate the long-term recursive capability of the model under deployment-oriented information constraints, this study adopted a strict recursive closed-loop multi-horizon forecasting protocol. Unlike the idealized evaluation setting in which future driving variables are known, strict recursive closed-loop forecasting does not use observed future greenhouse air temperature or observed future exogenous variables during inference. The model can access only historical observations before the forecast origin. During rolling forecasting, the simulated input sequence evolves using the model’s own predictions and the exogenous-variable generator, thereby avoiding information leakage and ensuring causal and fair evaluation.

**Figure 3:**
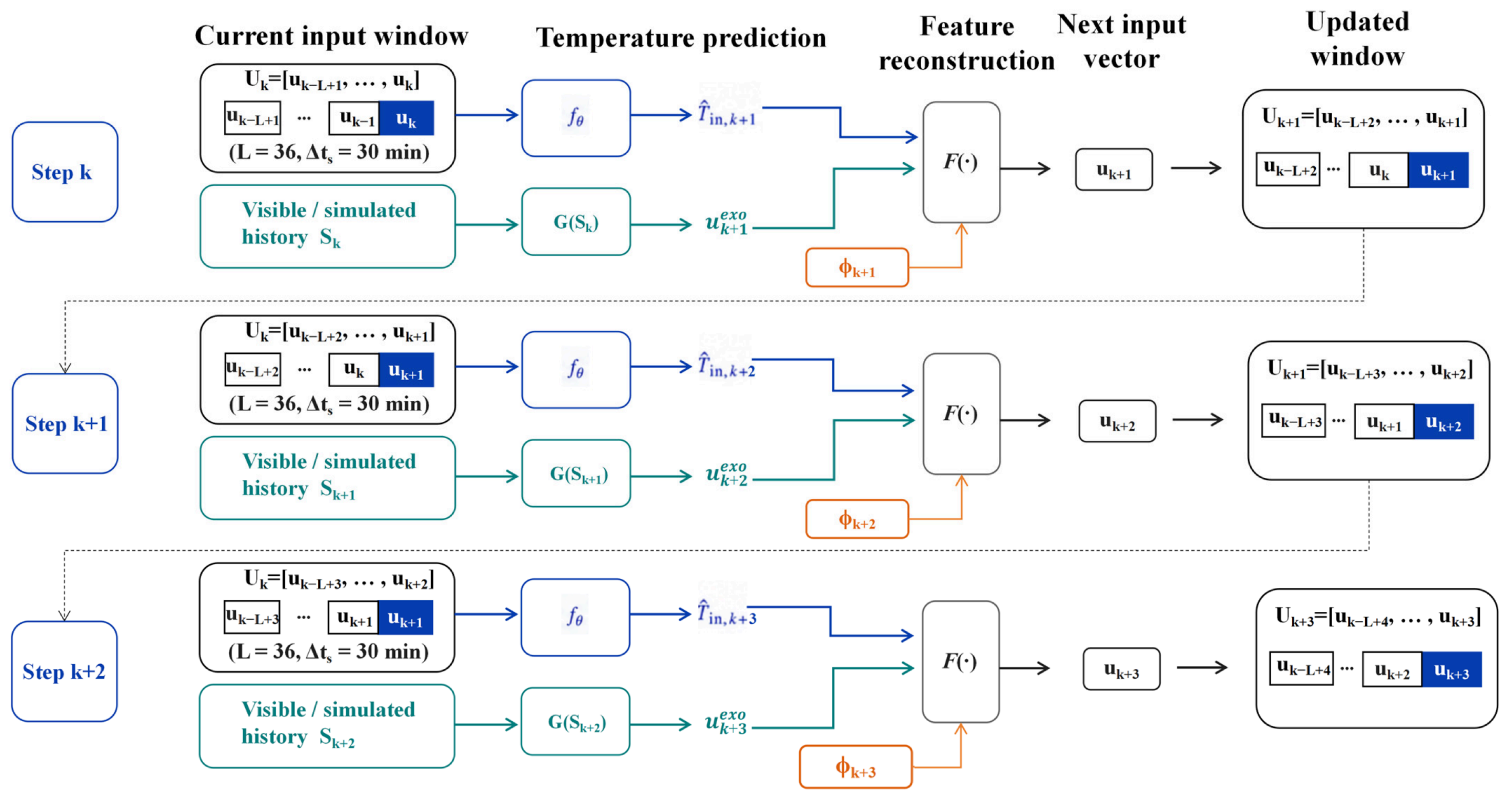
Schematic diagram of strict recursive closed-loop forecasting. Blue boxes and arrows represent the temperature prediction model and predicted-temperature feedback; green boxes and arrows represent the exogenous-variable generator and generated exogenous inputs; orange boxes and arrows represent time-periodic and derived features; and gray boxes represent feature reconstruction. Dashed arrows indicate the recursive transfer of the updated input window to the next forecasting step.

The closed-loop forecasting process is shown in Figure 3. Let the forecast origin be k_0_, the historical window length be L = 36, and the current rolling step be s. The end of the current simulated sequence is then k = k_0_ + s − 1. The model first uses the current historical input window U_k_ to predict the greenhouse air temperature at the next sampling time, denoted as $f_{\theta }(U_{k})$. Meanwhile, the exogenous-variable generator uses the currently visible and historical simulated sequence S_k_ to generate the future driving variables, denoted as G(S_k_). The predicted temperature, generated exogenous variables, and time-periodic and derived features $\phi_{k+1}$ are then jointly passed to the feature reconstruction function to obtain the next-step input vector:

(11) $$u_{k+1}\;=\;F\left[f_{\theta }(U_{k}),G(S_{k}),\phi_{k+1}\right]$$

where $u_{k+1}$ is the reconstructed input feature vector at the next sampling time; F is the closed-loop input feature reconstruction function; $f_{\theta }$ is the trained temperature forecasting model; $U_{k}$ is the current historical input window; $f_{\theta }(U_{k})$ represents the predicted greenhouse air temperature ${\hat{T}}_{\mathrm{in},\;k+1}$; G is the exogenous-variable generator; $S_{k}$ is the currently visible historical or simulated sequence information; $G(S_{k})$ represents the generated future exogenous driving vector $u_{k+1}^{exo}$; and $\phi_{k+1}$ represents the time-periodic encodings and related derived features at the next sampling time.

At the k-th prediction step, the historical input window $U_{k}$ is fed into the temperature forecasting model $f_{\theta }$ to obtain the predicted greenhouse air temperature ${\hat{T}}_{in,k+1}$ at the next sampling time. At the same time, the exogenous-variable generator $G(S_{k})$ generates the next-step exogenous variables based on the visible historical or simulated sequence. The feature reconstruction module F then combines the predicted temperature, generated exogenous variables, and time features to construct the next-step input vector $u_{k+1}$ Subsequently, $u_{k+1}$ is fed back into the input window to form the updated window $u_{k+1}$, which is used for the next prediction step. This process is recursively repeated over the 6 h, 24 h, and 48 h forecasting horizons without using observed future greenhouse air temperature or observed future exogenous variables.

#### 2.4.2. Exogenous-Variable Generator

Strict recursive closed-loop rolling forecasting requires that future exogenous variables should not be directly provided by observed values in the test set. Therefore, an exogenous-variable generator was constructed to provide future driving variables for the temperature forecasting model. The generated variables included outdoor temperature, outdoor relative humidity, solar radiation, wind speed, soil moisture, CO_2_ concentration, and indoor relative humidity. VPD was treated as a derived variable determined jointly by temperature and humidity. It was recalculated during feature reconstruction using the predicted temperature and generated humidity, rather than being directly read as an independent future exogenous variable.

For any exogenous variable z, the generator adopted a one-step linear prediction form. Its input features included the current value z_k_, first-order difference $\Delta z_{k}$, diurnal lag term $z_{k-48}$, and sine and cosine encodings of hour of day and day of year. Because the data sampling interval was 30 min, 48 sampling steps corresponded to 24 h. Therefore, $z_{k-48}$ was used to represent the diurnal periodicity of the exogenous variable. The input feature vector and one-step generation process were defined as follows:

(12) $$q_{z,k}={\left[1,\;z_{k},\;\Delta z_{k},\;z_{k-48},\;\sin\left(\frac{2\pi h_{k}}{24}\right),\;\cos\left(\frac{2\pi h_{k}}{24}\right),\;\sin\left(\frac{2\pi d_{k}}{365}\right),\;\cos\left(\frac{2\pi d_{k}}{365}\right)\right]}^{T}$$

(13) $${\hat{z}}_{k+1}=W_{z}^{T}q_{z,k}$$

where $q_{z,k}$ is the generator input feature vector for exogenous variable *z* at the k-th sampling point; $z_{k}$ is the current value of the exogenous variable; $\Delta z_{k}\;=\;z_{k}-\;z_{k-1}$ is the first-order difference; $z_{k-48}$ is the diurnal lag term; $h_{k}$ is the current hour; $d_{k}$ is the day of year; $w_{z}$ is the parameter vector of the linear generator for variable z; and ${\hat{z}}_{k+1}$ is the generated value of the exogenous variable at the next sampling time.

To assess the limitations of the linear generator, a nonlinear residual gradient-boosted regression-tree generator (Residual-GBRT) was constructed as a diagnostic control. It retained the linear prediction as the backbone and learned the remaining residual from multivariate current values, first-order differences, 24 h lags, and time-cycle encodings under the same data split and recursive forecasting protocol. Because the primary temperature models were developed under the recursive input distribution produced by the linear generator, Residual-GBRT was not substituted into the main forecasting protocol.

#### 2.4.3. Evaluation of Exogenous-Variable Generation Error and Error Propagation

To analyse the sources of strict recursive closed-loop forecasting error, the generation errors of the exogenous-variable generator were evaluated, and the relationship between exogenous-variable generation errors and greenhouse air temperature prediction errors was further analysed. Exogenous-variable generation errors were evaluated using MAE, RMSE, Bias, and normalized RMSE (NRMSE). The calculations of MAE and RMSE were consistent with those used for temperature prediction errors. Bias represents the mean deviation between generated and observed values and was used to determine whether the generator tended to overestimate or underestimate a variable. NRMSE represents the ratio of RMSE to the observed range of the corresponding variable and was used to compare relative error levels across variables with different units. Bias and NRMSE were calculated as follows:

(14) $$\begin{array}{c} Bias_{z}\;=\;\frac{1}{m}\sum_{i=1}^{m}\left({\hat{z}}_{i}-z_{i}\right) \end{array}$$

(15) $$\begin{array}{c} NRMSE_{z}=\frac{\sqrt{\frac{1}{m}\sum_{i=1}^{m}{\left({\hat{z}}_{i}-z_{i}\right)}^{2}}}{z_{\max}-z_{\min}} \end{array}$$

where m is the number of exogenous-variable generation samples; ${\hat{z}}_{i}$ and $z_{i}$ are the generated and observed values of the i-th sample, respectively; and $z_{max}$ and $z_{min}$ are the maximum and minimum observed values of the corresponding exogenous variable.

To further determine whether exogenous-variable errors were associated with greenhouse air temperature prediction errors, the Spearman correlation coefficient between the absolute generation error of each exogenous variable and the absolute prediction error of greenhouse air temperature was calculated:

(16) $$\rho_{z,\;H}\;=\;\rho_{s}\left(\left|{\hat{z}}_{H}-z_{H}\right|,\left|{\hat{T}}_{\mathrm{in},\;H}-T_{\mathrm{in},\;H}\right|\right)$$

where $\rho_{z,H}$ is the Spearman correlation coefficient between the generation error of exogenous variable *z* and the greenhouse air temperature prediction error under forecasting horizon H; $\rho_{s}$ denotes the Spearman rank correlation function; $\left|{\hat{z}}_{H}-z_{H}\right|$ is the absolute generation error of the exogenous variable; and $\left|{\hat{T}}_{in,H}-T_{in,H}\right|$ is the absolute prediction error of greenhouse air temperature. The statistical significance of each Spearman correlation coefficient was evaluated using a two-sided test. Because eight exogenous variables were examined under three forecasting horizons, the resulting 24 *p*-values were adjusted using the Benjamini–Hochberg false discovery rate procedure to control for multiple comparisons. An FDR-adjusted *p*-value below 0.05 was considered statistically significant. By comparing the correlation coefficients under 6 h, 24 h, and 48 h forecasting horizons, the potential effect of exogenous-variable generation errors on the accumulation of greenhouse air temperature prediction errors can be analysed.

### 2.5. Ablation Experiment on Recursive Forecasting Protocols

To verify the necessity of the strict recursive closed-loop forecasting protocol and to analyse the effect of future exogenous-variable acquisition methods on multi-horizon temperature forecasting, ablation experiments on closed-loop forecasting protocols were designed based on the trained LNN model. All three protocols did not use observed future greenhouse air temperature. They differed only in the way future exogenous variables were obtained.

Semi-closed-loop forecasting used predicted greenhouse air temperature as feedback to update the input window, while future exogenous variables were taken from observed values in the test set. This protocol represented an ideal exogenous-driving condition and was used to evaluate the recursive forecasting capability of the temperature model under accurate future exogenous inputs. Strict recursive closed-loop forecasting also used predicted greenhouse air temperature as feedback, but future exogenous variables were recursively produced by the exogenous-variable generator. This protocol did not use observed future greenhouse air temperature or observed future exogenous variables and served as the main evaluation protocol for multi-horizon forecasting in this study. The persistence baseline for exogenous variables also used predicted greenhouse air temperature as feedback, but future exogenous variables were kept equal to the most recent visible values at the forecast origin. This baseline was used to evaluate the error level under a simple exogenous-variable assumption. The three forecasting protocols are shown in Table 2.

The ablation experiments were conducted over 6 h, 24 h, and 48 h forecasting horizons. Because the data sampling interval was 30 min, the three horizons corresponded to 12, 48, and 96 rolling prediction steps, respectively. The three protocols used the same test origins and evaluation procedure, and MAE and RMSE were calculated over all rolling steps within each forecasting window. By comparing semi-closed-loop forecasting with strict recursive closed-loop forecasting, the effect of exogenous-variable generation errors on closed-loop forecasting performance was analysed. By comparing strict recursive closed-loop forecasting with the persistence baseline, whether the exogenous-variable generator could reduce long-horizon prediction errors compared with a simple persistence strategy was evaluated.

In real greenhouse deployment, future outdoor meteorological variables may be obtained from weather forecasts, while some greenhouse-specific variables may depend on control schedules or management-event records. In the present study, however, historical weather-forecast products synchronized with the sensor dataset were not archived during data collection. Therefore, a quantitative forecast-based exogenous protocol could not be implemented without introducing additional external data sources that were not part of the original experimental record. The present ablation experiment therefore focused on three information conditions: ideal observed exogenous inputs, generated exogenous inputs, and persistence exogenous inputs.

## 3. Results

This section presents the results of strict recursive closed-loop forecasting for greenhouse air temperature. First, under the same data partitioning, input variables, historical window length, and evaluation metrics, the performance of LNN, LSTM, GRU, and XGBoost was compared in one-step prediction and in strict recursive closed-loop rolling forecasting over 6 h, 24 h, and 48 h horizons. Then, the prediction trajectories and predicted–observed scatter distributions were analysed to evaluate the ability of the models to track the dynamic variation of greenhouse air temperature and to characterize long-horizon recursive error patterns. On this basis, the generation errors of the exogenous-variable generator were further evaluated under different forecasting horizons, and the relationships between exogenous-variable errors and greenhouse air temperature prediction errors were analysed. Finally, ablation experiments on closed-loop forecasting protocols were conducted to examine the effects of different future exogenous-variable acquisition methods on the multi-horizon forecasting performance of the LNN.

### 3.1. Prediction Model Performance Analysis

Under the same data partitioning, input variables, historical window length, and evaluation metrics, LNN, LSTM, GRU, and XGBoost were evaluated in one-step prediction and in strict recursive closed-loop forecasting over 6 h, 24 h, and 48 h horizons. The results are shown in Table 3. In one-step prediction, the models used observed historical sequences to predict greenhouse air temperature at the next sampling time. In strict recursive closed-loop rolling forecasting, observed future greenhouse air temperature and observed future exogenous variables were not used during inference. Instead, future inputs were recursively constructed through predicted-temperature feedback and the exogenous-variable generator.

The Friedman tests showed significant overall differences among the four models for both MAE and RMSE at all forecasting horizons (*p* < 0.001). The Holm-adjusted pairwise comparisons further showed that the LNN achieved significantly lower one-step prediction errors than the other models, whereas the LSTM achieved significantly lower errors than the other models under the 6 h, 24 h, and 48 h strict recursive closed-loop forecasting horizons (*p*_adj_ < 0.05).

**Figure 4:**
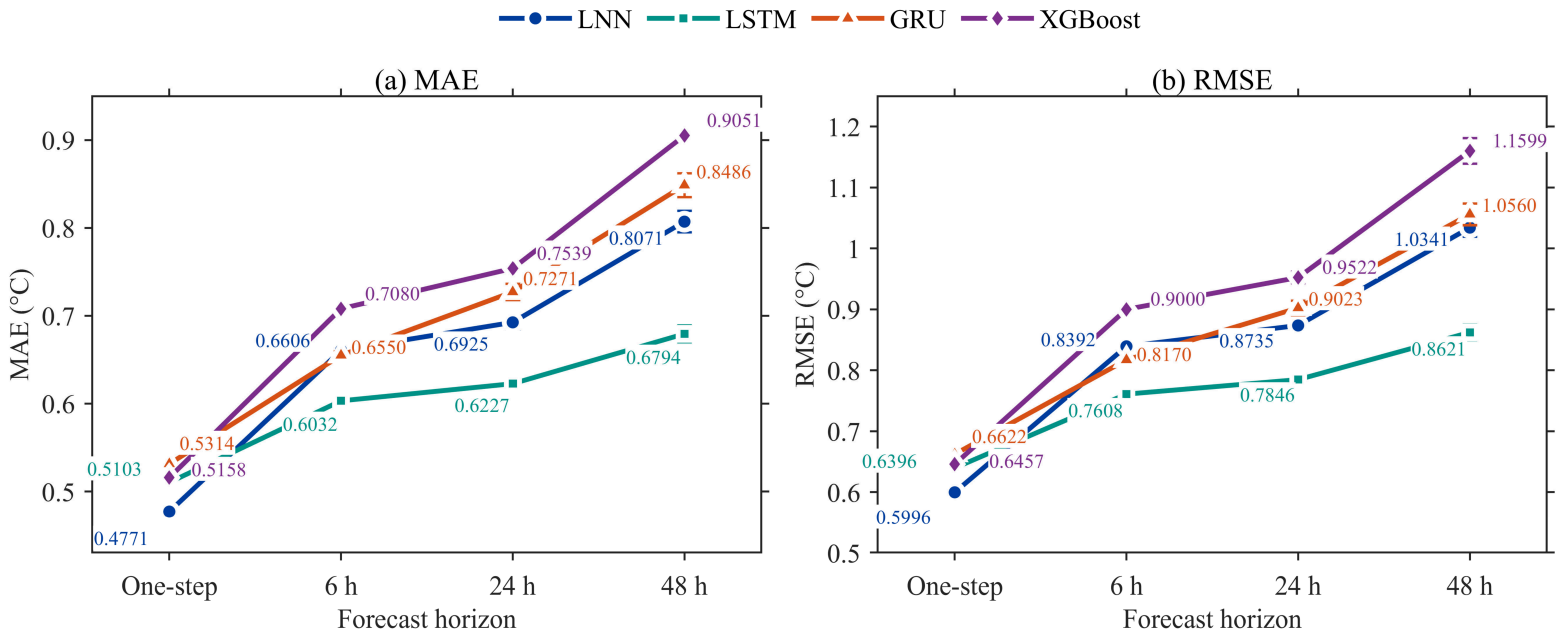
Error comparison of different models under one-step prediction and strict recursive closed-loop forecasting: (**a**) MAE changes in LNN, LSTM, GRU, and XGBoost under one-step prediction and strict recursive closed-loop rolling forecasting over 6 h, 24 h, and 48 h horizons; (**b**) RMSE changes in the four models under the same forecasting settings. “One-step” denotes one-step prediction, whereas 6 h, 24 h, and 48 h denote strict recursive closed-loop rolling forecasting horizons.

Across the 10 repeated runs, the LNN achieved the lowest one-step prediction errors, with an MAE of 0.4771 ± 0.0009 °C and an RMSE of 0.5996 ± 0.0010 °C. Compared with LSTM, GRU, and XGBoost, the mean MAE of the LNN was reduced by 6.51%, 10.22%, and 7.50%, respectively, and the corresponding RMSE reductions were 6.25%, 9.45%, and 7.14%. However, the model ranking changed under strict recursive closed-loop forecasting. LSTM achieved the lowest mean MAE and RMSE at the 6 h, 24 h, and 48 h horizons. Compared with LNN, the MAE of LSTM was lower by 8.70%, 10.08%, and 15.82% at the three horizons, respectively, while its RMSE was lower by 9.34%, 10.36%, and 16.63%, respectively. Prediction errors generally increased with the forecasting horizon, reflecting the accumulation of predicted-temperature feedback errors and exogenous-variable generation errors during recursion. From the 6 h to the 48 h horizon, the MAE and RMSE of the LNN increased by 22.16% and 23.22%, respectively, whereas the corresponding increases for LSTM were 12.63% and 13.31%. Thus, LSTM exhibited a slower horizon-dependent increase in recursive prediction error under the same evaluation protocol. These results confirm that one-step prediction accuracy and long-horizon recursive stability should be evaluated separately. The corresponding changes in MAE and RMSE across the different forecasting horizons are shown in Figure 4.

**Figure 5:**
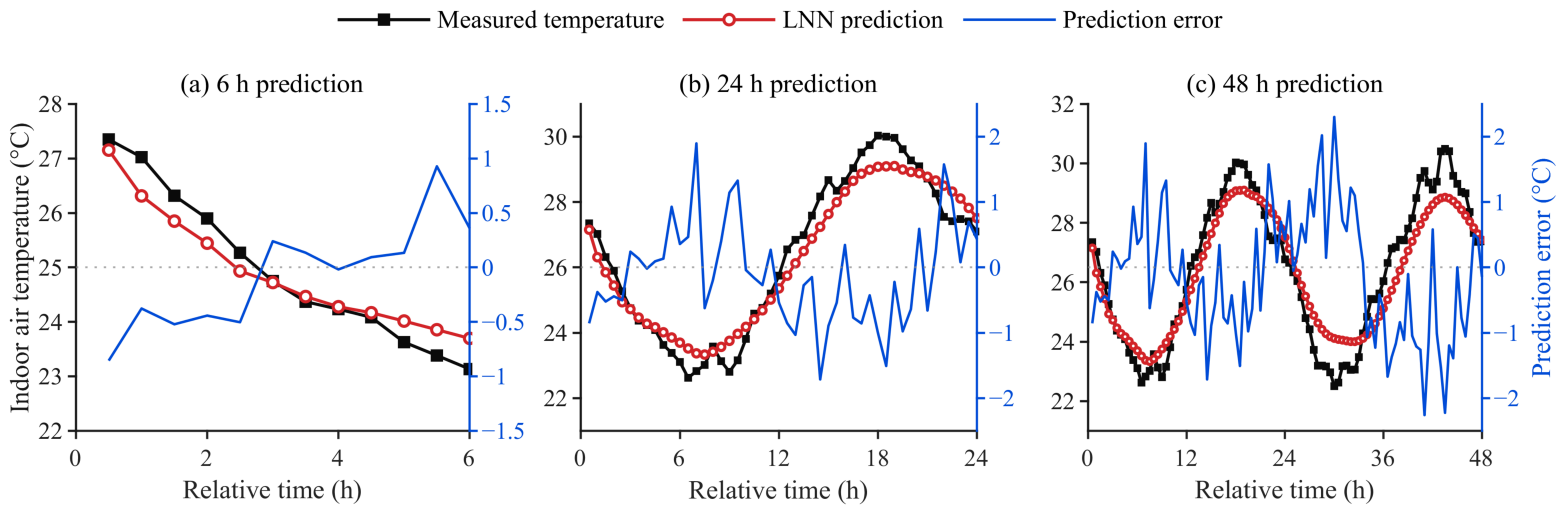
Strict recursive closed-loop forecasting trajectories and error changes in the LNN: (**a**) LNN forecasting trajectory and prediction error under the 6 h horizon; (**b**) LNN forecasting trajectory and prediction error under the 24 h horizon; (**c**) LNN forecasting trajectory and prediction error under the 48 h horizon.

To further analyse the strict recursive closed-loop forecasting performance of the LNN during continuous temperature variation, the rolling prediction trajectories over the 6 h, 24 h, and 48 h horizons were visualized, as shown in Figure 5. Overall, the LNN was able to follow the main variation trend of greenhouse air temperature under different forecasting horizons. Within the 6 h horizon, the predicted curve was generally consistent with the observed curve, and the errors were mainly reflected in local amplitude deviations. As the forecasting horizon was extended to 24 h and 48 h, the diurnal peaks and valleys became more pronounced, and the prediction error range increased accordingly. In particular, the predicted curve showed a smoothing tendency at temperature peaks, valleys, and rapid transition stages, indicating that the LNN could maintain the overall temperature trend but still had limitations in capturing local extrema and abrupt changes. This phenomenon may be related to the recursive use of predicted temperatures and generated exogenous variables in the strict recursive closed-loop process. Once the generation errors of key driving variables, such as outdoor temperature and solar radiation, enter the input window, they can affect subsequent temperature predictions and lead to accumulated trajectory deviations over longer horizons.

**Figure 6:**
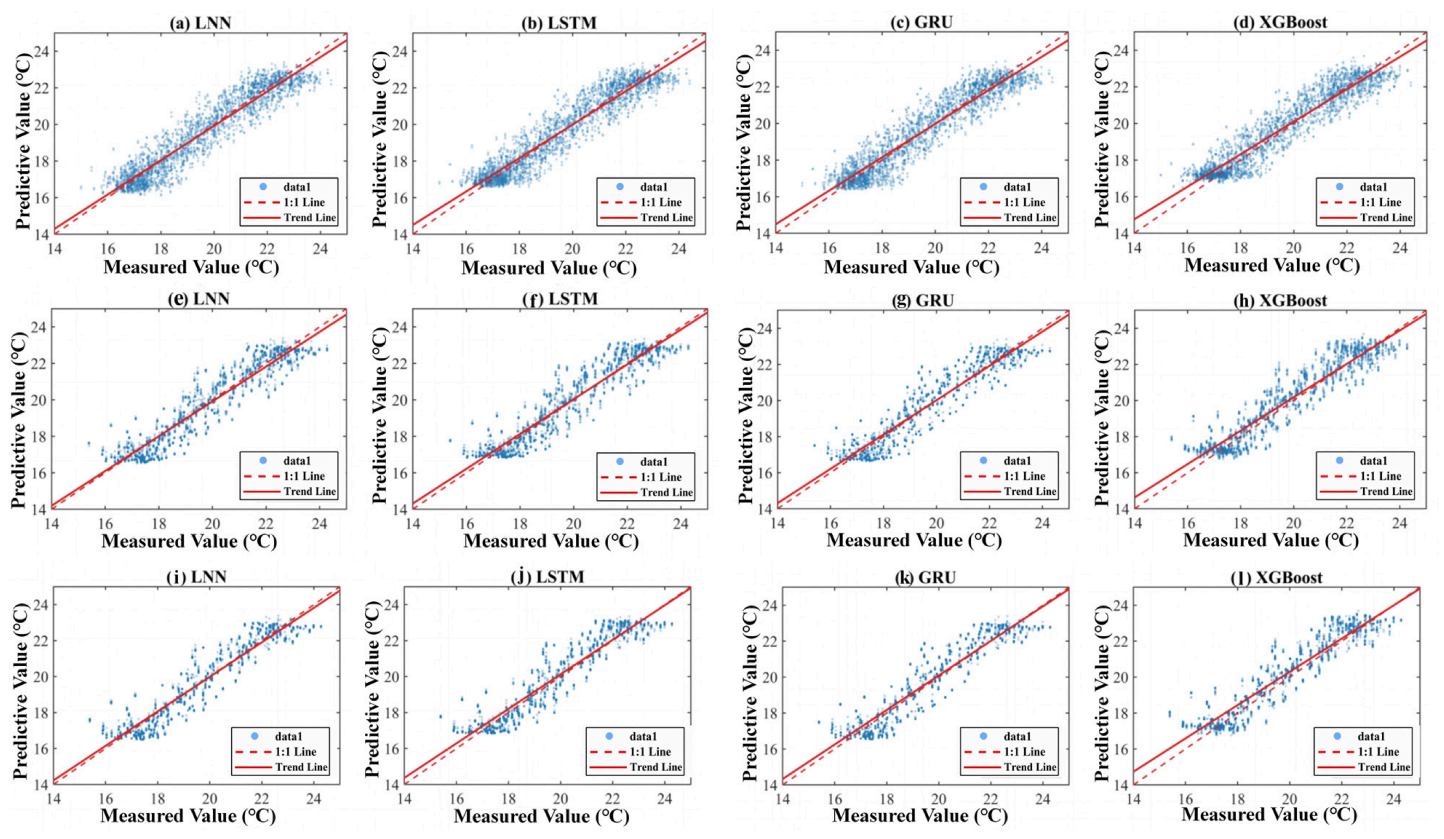
Scatter distributions of predicted and observed values under different forecasting horizons: (**a**–**d**) Prediction scatter plots of LNN, LSTM, GRU, and XGBoost under the 6 h horizon, respectively; (**e**–**h**) prediction scatter plots of LNN, LSTM, GRU, and XGBoost under the 24 h horizon, respectively; (**i**–**l**) prediction scatter plots of LNN, LSTM, GRU, and XGBoost under the 48 h horizon, respectively. Each point represents one rolling prediction result under the corresponding forecasting horizon.

To evaluate the overall prediction consistency of the models across all test samples, full-sample predictions were further analysed for each model, as shown in Figure 6.

As shown in Figure 6, under the 6 h forecasting horizon, the scatter points of all four models were generally concentrated near the 1:1 reference line, indicating good agreement between predicted and observed values. When the forecasting horizon was extended to 24 h and 48 h, the scatter distributions became more dispersed, and deviations increased in both high- and low-temperature ranges. This pattern reflected the accumulation of closed-loop recursive errors at the full-sample scale. Overall, all four models showed increased scatter dispersion as the forecasting horizon extended. LSTM showed the most stable full-sample prediction consistency under the strict recursive closed-loop setting, whereas the LNN maintained better consistency than GRU and XGBoost at the 24 h and 48 h horizons but did not exceed LSTM.

### 3.2. Exogenous-Variable Generation Error and Propagation Analysis

During strict recursive closed-loop forecasting, future exogenous variables cannot be directly provided by observed values in the test set. Instead, they must be recursively produced by the exogenous-variable generator. Because different exogenous variables have different levels of association with greenhouse air temperature, their generation errors may have different effects on the final temperature prediction results. Therefore, this section first analyses the correlations between each exogenous variable and greenhouse air temperature to identify the key driving variables most closely related to temperature variation. The generation performance of the exogenous-variable generator is then evaluated under different forecasting horizons. Finally, the correlations between exogenous-variable generation errors and greenhouse air temperature prediction errors are analysed to characterize how exogenous errors propagate into temperature prediction errors.

#### 3.2.1. Correlation Analysis Between Exogenous Variables and Greenhouse Air Temperature

**Figure 7:**
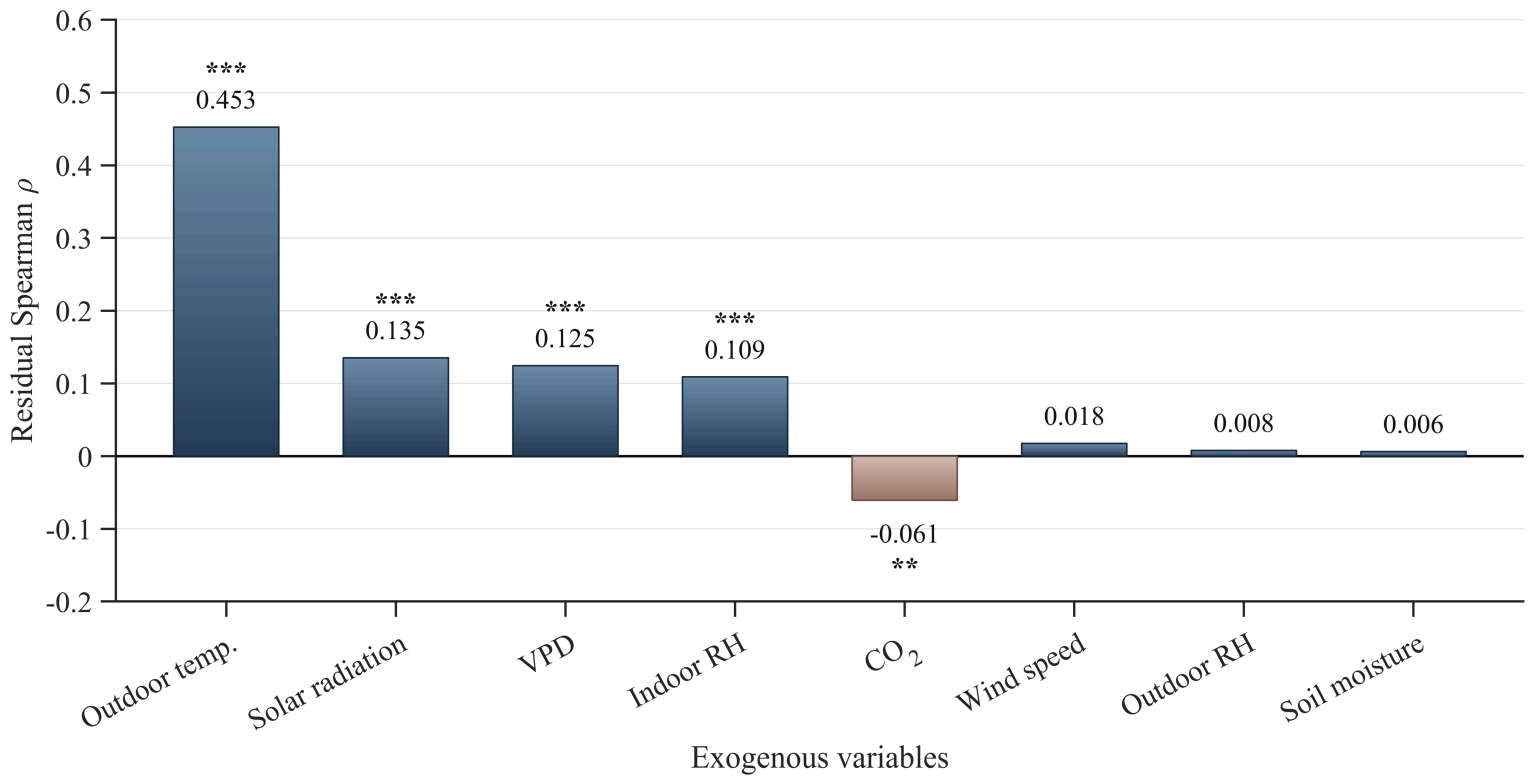
Correlation analysis between exogenous variables and greenhouse air temperature: The bar length represents the correlation strength. *** indicates *p* < 0.001, ** indicates *p* < 0.01, and variables without significance markers are not statistically significant.

In strict recursive closed-loop rolling forecasting, future exogenous variables are recursively generated and then used as inputs for subsequent temperature prediction. Therefore, the degree to which different exogenous variables are associated with greenhouse air temperature is an important prerequisite for judging whether their generation errors may affect temperature prediction. If an exogenous variable has only a weak relationship with greenhouse air temperature, even a large generation error may not substantially amplify the temperature prediction error. In contrast, if an exogenous variable is closely related to greenhouse air temperature, its generation error is more likely to shift the predicted temperature trajectory after entering the closed-loop input window. Based on this consideration, the Spearman correlation coefficients between greenhouse air temperature and outdoor temperature, outdoor relative humidity, solar radiation, wind speed, soil moisture, CO_2_ concentration, indoor relative humidity, and VPD were calculated to identify key exogenous variables associated with greenhouse air temperature variation. The results are shown in Figure 7.

As shown in Figure 7, the correlations between different exogenous variables and short-term variations in greenhouse air temperature differed substantially. Outdoor temperature showed the strongest correlation with greenhouse air temperature, with a Spearman coefficient of ρ = 0.453 (p < 0.001). Solar radiation, VPD, and indoor relative humidity showed weak but significant positive correlations with greenhouse air temperature, with correlation coefficients of 0.135, 0.125, and 0.109, respectively, all reaching the *p* < 0.001 significance level. CO_2_ concentration showed a weak negative correlation with greenhouse air temperature, with ρ=−0.061 (p=0.0011). Wind speed, outdoor relative humidity, and soil moisture showed weak correlations that did not reach statistical significance.

These results show that exogenous variables differed in their relevance to greenhouse air temperature prediction. Outdoor temperature had the closest relationship with short-term greenhouse air temperature variation. Although the correlation of solar radiation was weaker, it remained statistically significant. Therefore, under strict recursive closed-loop long-horizon forecasting, the generation accuracy of outdoor temperature and solar radiation deserves particular attention.

#### 3.2.2. Exogenous-Variable Generation Performance Analysis

In strict recursive closed-loop forecasting, the model cannot directly use observed future exogenous variables and must rely on the exogenous-variable generator to recursively produce future driving variables. Therefore, the quality of exogenous-variable generation directly affects the stability of multi-horizon temperature forecasting. To analyse changes in exogenous-driving errors under different forecasting horizons, MAE, RMSE, Bias, and NRMSE were calculated for outdoor temperature, outdoor relative humidity, solar radiation, wind speed, soil moisture, CO_2_ concentration, indoor relative humidity, and VPD under the 6 h, 24 h, and 48 h horizons. The results are shown in Table 4.

As shown in Table 4, generation errors differed markedly among exogenous variables, and the errors of most variables increased as the forecasting horizon was extended. Solar radiation had relatively high absolute errors, with an MAE of 108.09 μmol m^−2^ s^−1^ and an RMSE of 175.51 μmol m^−2^ s^−1^ at the 48 h horizon. This result shows that the linear exogenous-variable generator had limited ability to reconstruct radiation peaks and short-term fluctuations. The MAE of outdoor temperature increased from 1.38 °C at 6 h to 1.96 °C at 48 h, and the MAE of outdoor relative humidity increased from 3.38% RH to 4.65% RH. These results indicate continuous bias accumulation in meteorological boundary conditions during long-horizon recursion. The MAE of indoor relative humidity increased from 3.75% RH to 4.69% RH, showing that estimation errors in the internal hydrothermal state of the greenhouse also increased as the forecasting horizon became longer.

**Figure 8:**
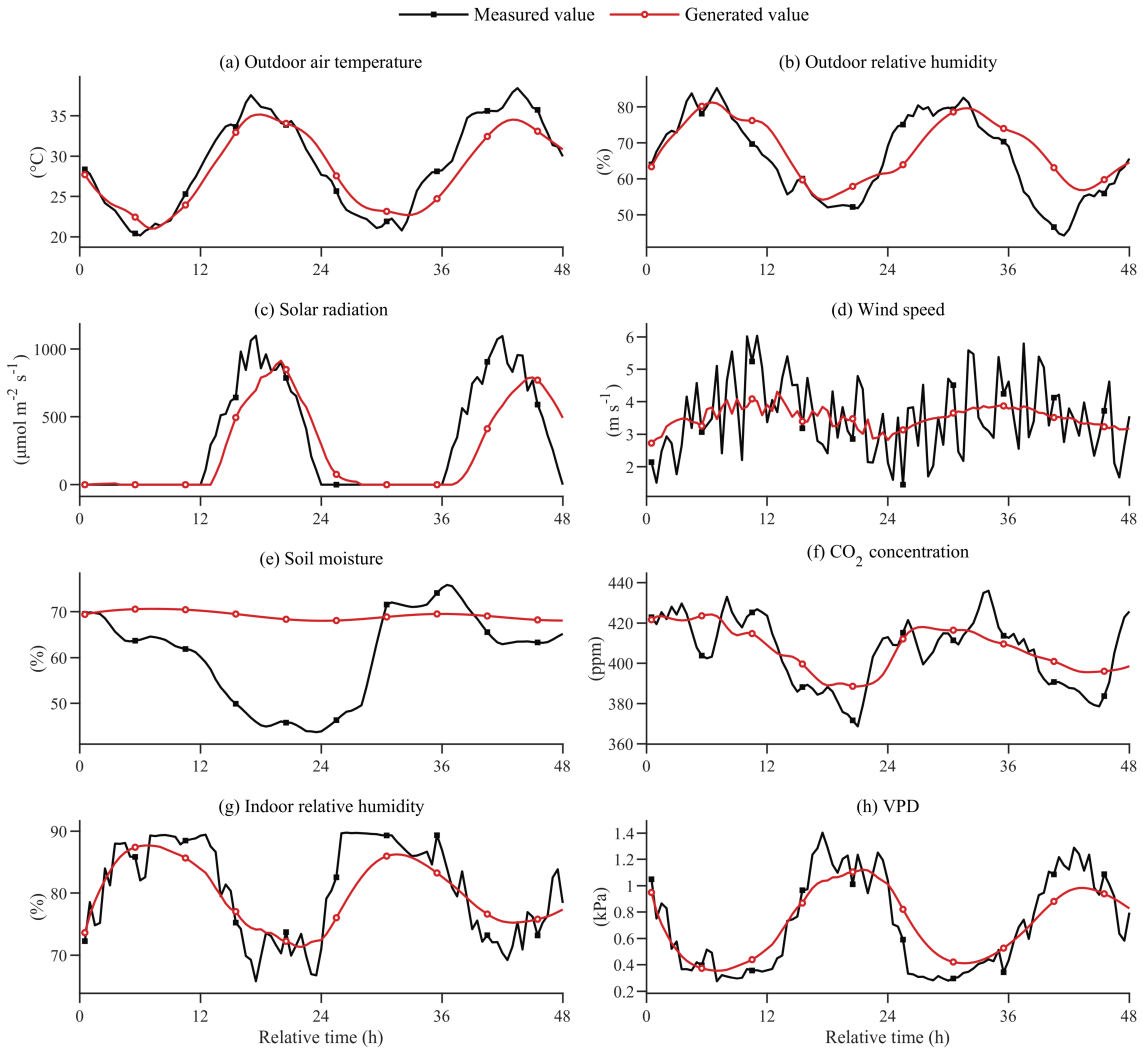
Comparison between observed and generated values of major exogenous variables during a typical 48 h forecasting process: (**a**) Outdoor temperature. (**b**) Outdoor relative humidity. (**c**) Solar radiation. (**d**) Wind speed. (**e**) Soil moisture. (**f**) CO_2_ concentration. (**g**) Indoor relative humidity. (**h**) VPD.

A typical 48 h forecasting process was further selected to compare the observed and generated values of the main exogenous variables, as shown in Figure 8. The exogenous-variable generator generally tracked the main trends of variables such as outdoor temperature and outdoor relative humidity. This indicates that the linear generator based on the current value, first-order difference, diurnal lag term, and time-periodic encodings captured part of the periodic variation in exogenous variables. However, for variables with stronger fluctuations or stronger effects of management events, such as solar radiation, wind speed, and soil moisture, deviations remained between generated and observed values. Solar radiation was strongly affected by diurnal cycles, weather fluctuations, and shading changes, and the generated curve did not fully capture peaks and rapid changes. Wind speed showed strong random disturbances, making it difficult for the generated values to follow short-term fluctuations. Soil moisture was jointly affected by irrigation, evaporation, and crop water uptake, and local abrupt changes and long-term trend shifts were difficult to reconstruct accurately using a simple linear generator. These results show that the exogenous-variable generator provided the future driving variables required for strict recursive closed-loop forecasting, but its tracking ability differed among variables.

**Figure 9:**
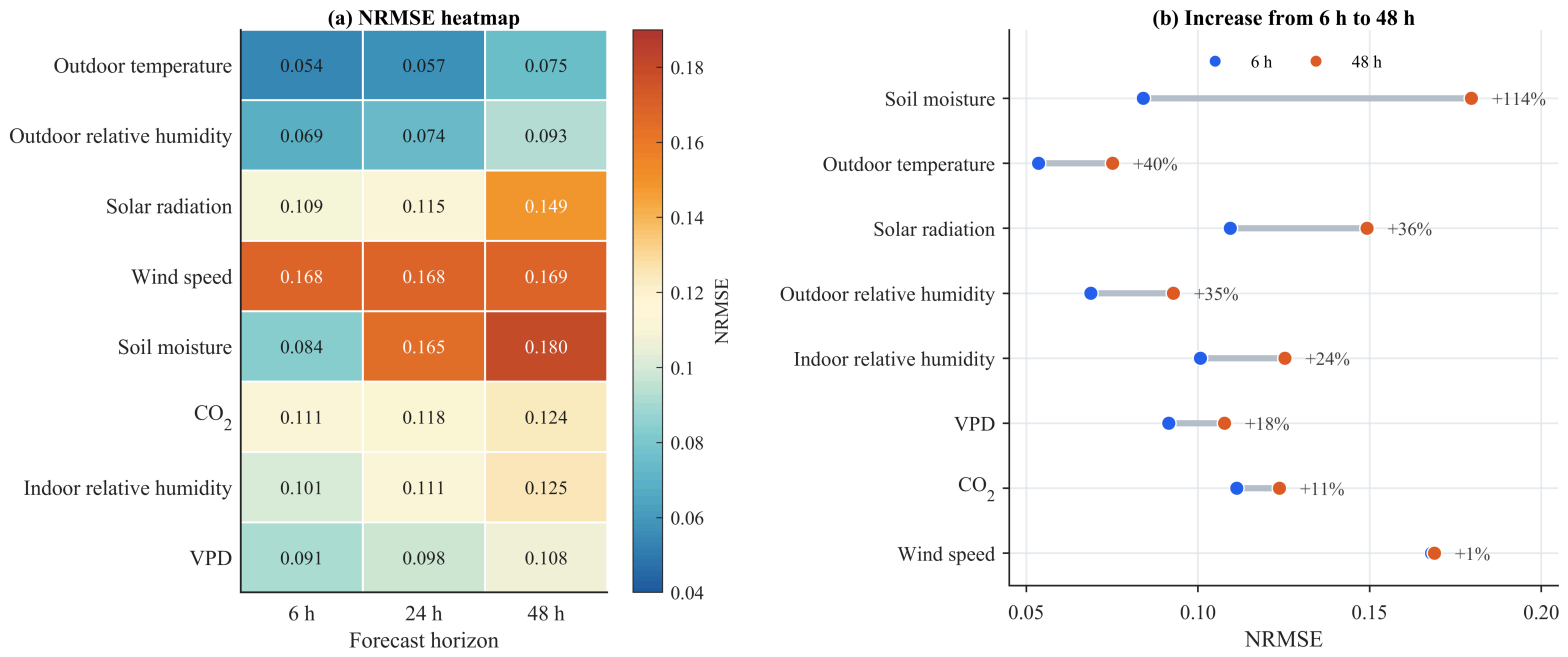
NRMSE of exogenous-variable generation errors and their relative increase from 6 h to 48 h: (**a**) NRMSE heatmap of exogenous-variable generation errors under 6 h, 24 h, and 48 h forecasting horizons; (**b**) relative increase in NRMSE from the 6 h to the 48 h forecasting horizon.

The relative error changes in different variables are shown in Figure 9. According to the NRMSE results, soil moisture showed the most pronounced increase in relative error, rising from 0.084 at 6 h to 0.180 at 48 h. This result indicates that soil moisture was more prone to trend shifts during long-horizon recursion. The NRMSE values of wind speed were 0.168, 0.169, and 0.169 under the 6 h, 24 h, and 48 h horizons, respectively. Although wind speed remained at a relatively high error level, it changed little as the horizon increased. This indicates that wind speed had strong inherent random fluctuations and was difficult to generate even in the short term, but it did not show obvious horizon-dependent error accumulation. The errors of CO_2_ concentration and VPD also increased as the forecasting horizon was extended, but the increases were relatively limited.

Overall, the exogenous-variable generator captured the main variation trends of some exogenous variables and provided the future exogenous variables required for strict recursive closed-loop forecasting. However, the generation errors showed clear variable-dependent differences. Solar radiation, outdoor temperature, soil moisture, and indoor relative humidity were the variables with more prominent generation errors under long forecasting horizons.

The diagnostic comparison between the linear generator and Residual-GBRT is presented in Supplementary Figure S1 and Supplementary Table S3. Residual-GBRT reduced both MAE and RMSE in 15 of the 21 variable–horizon combinations. The largest improvement occurred for solar radiation at the 48 h horizon, where MAE and RMSE decreased by 22.54% and 16.95%, respectively. Wind-speed errors decreased modestly, while improvements were also observed for indoor and outdoor relative humidity and CO_2_ concentration. In contrast, outdoor-temperature and soil-moisture errors were not consistently reduced. These results confirm that the linear generator underrepresented some nonlinear variations, particularly solar-radiation dynamics, but also show that nonlinear residual correction did not provide a universal advantage across all exogenous variables.

#### 3.2.3. Propagation Analysis of Exogenous-Variable Errors

**Figure 10:**
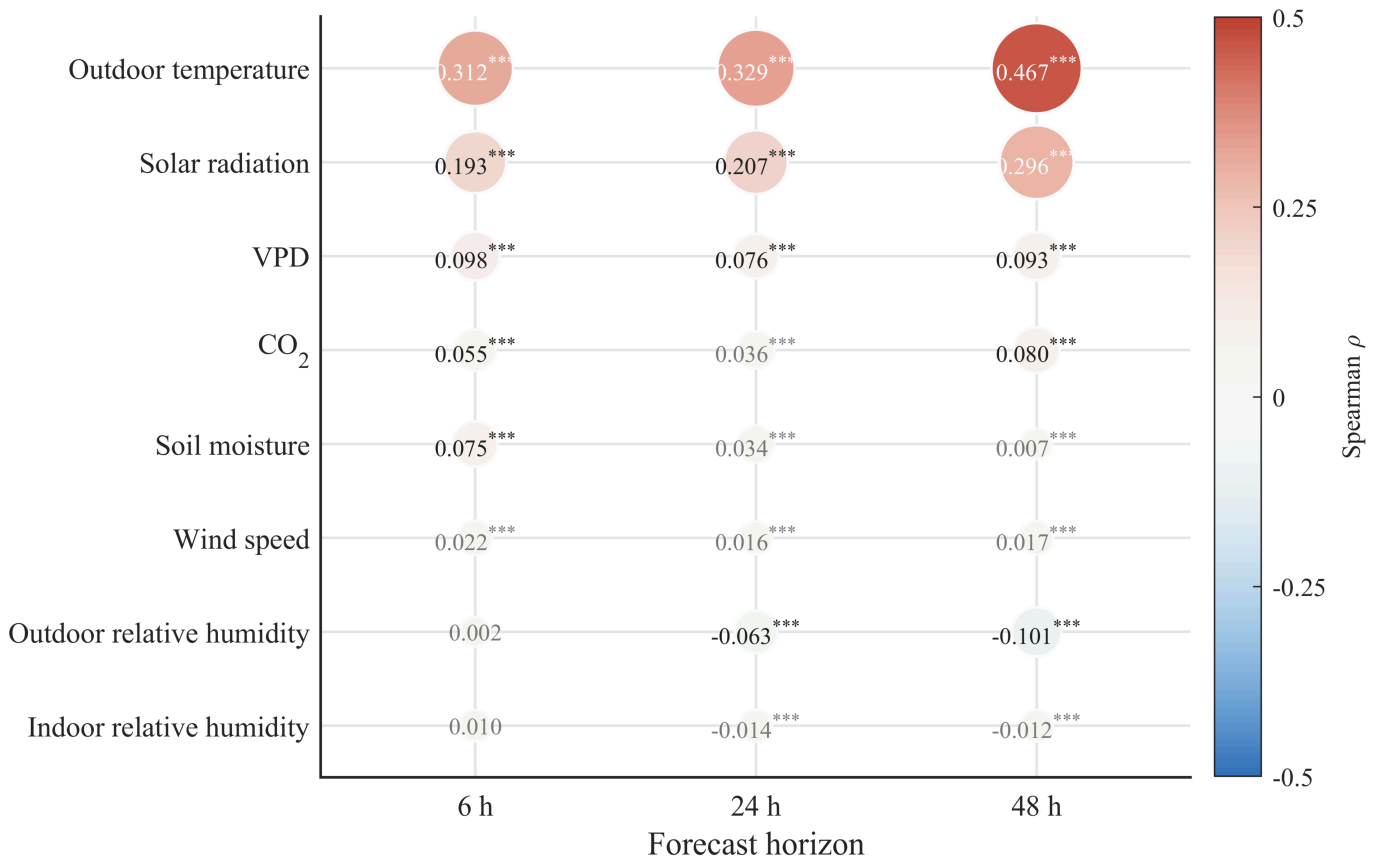
Spearman correlation heatmap between absolute exogenous-variable generation errors and absolute greenhouse air temperature prediction errors: under the 6 h, 24 h, and 48 h forecasting horizons. Values in the circles represent Spearman correlation coefficients. *** indicate a Benjamini–Hochberg FDR-adjusted *p* < 0.001; correlations without significance markers are not statistically significant.

A large exogenous-variable generation error does not necessarily mean that it will substantially affect the greenhouse air temperature prediction result. Some variables may have large generation errors, but if their direct driving effect on greenhouse air temperature is weak within the current forecasting horizon, they may not noticeably amplify the temperature prediction error. In contrast, thermal boundary and energy input variables, such as outdoor temperature and solar radiation, may have a more direct effect on temperature trajectory shifts even when their errors are not the largest among all variables. Therefore, after evaluating exogenous-variable generation performance, the Spearman correlation coefficients between the absolute generation error of each exogenous variable and the absolute prediction error of greenhouse air temperature were calculated. The results are shown in Figure 10.

As shown in Figure 10, outdoor-temperature generation error was significantly and positively correlated with greenhouse air temperature prediction error at the 6 h, 24 h, and 48 h horizons, with Spearman correlation coefficients of 0.3119, 0.3294, and 0.4674, respectively (FDR-adjusted *p* < 0.001). Solar-radiation generation error also showed significant positive correlations at the three forecasting horizons, with coefficients of 0.1928, 0.2070, and 0.2955, respectively (FDR-adjusted *p* < 0.001). The increasing correlation coefficients indicate that the generation errors of these two thermal-driving variables became more closely associated with greenhouse air temperature prediction deviations as the recursive forecasting horizon increased.

Several other variables also showed statistically significant correlations because of the large number of error observations; however, their correlation coefficients were generally small. For example, the absolute correlations for wind speed, soil moisture, CO_2_ concentration, indoor relative humidity, and VPD were mostly below 0.10. Therefore, statistical significance was interpreted together with correlation magnitude. Outdoor temperature showed the strongest and most consistent association with temperature prediction error, while solar radiation showed a weaker but stable positive association. These results indicate that outdoor temperature and solar radiation were the more practically relevant sources of propagated exogenous error under strict recursive closed-loop forecasting.

### 3.3. Ablation Experiment Results

**Figure 11:**
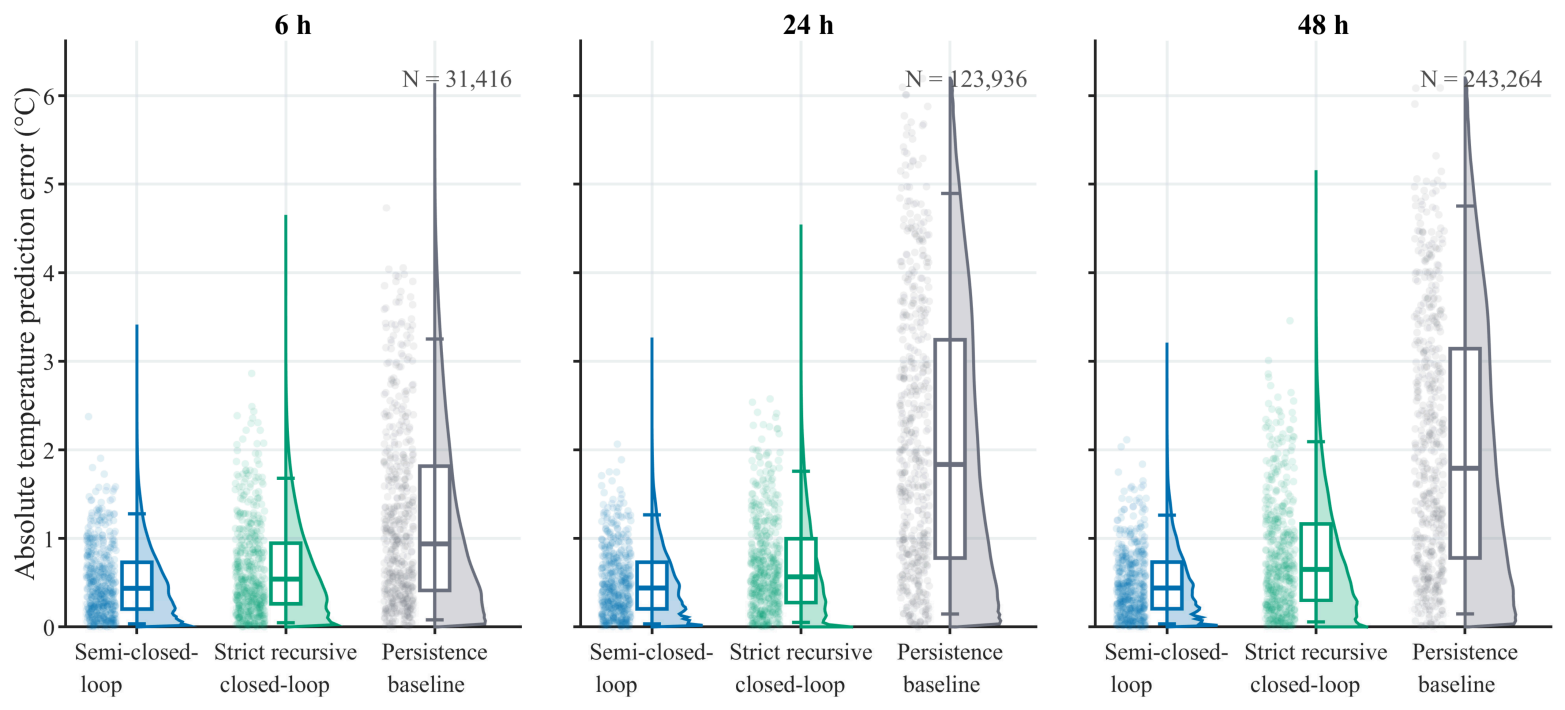
Distribution of absolute LNN prediction errors under different closed-loop forecasting protocols at the 6 h, 24 h, and 48 h forecasting horizons.

To verify the necessity of the strict recursive closed-loop forecasting protocol and to analyse the effect of future exogenous-variable acquisition methods on the multi-horizon forecasting performance of the LNN, three closed-loop forecasting protocols were designed based on the trained LNN model. All three protocols did not use observed future greenhouse air temperature, and all used predicted-temperature feedback to update the input window. The only difference among them was the source of future exogenous variables. In semi-closed-loop forecasting, future exogenous variables were taken from observed values in the test set. In strict recursive closed-loop forecasting, future exogenous variables were recursively generated by the exogenous-variable generator. In the persistence baseline for exogenous variables, future exogenous variables were kept equal to the most recent visible values at the forecast origin. The prediction errors of the LNN under different protocols are shown in Table 5, and the corresponding error changes are shown in Figure 11.

As shown in Table 5, the source of future exogenous variables had a clear effect on the closed-loop forecasting performance of the LNN. Semi-closed-loop forecasting achieved the lowest errors at the 6 h, 24 h, and 48 h horizons, with MAE/RMSE values of 0.5094/0.6422 °C, 0.5104/0.6425 °C, and 0.5107/0.6421 °C, respectively. The errors changed only slightly as the forecasting horizon increased, indicating that the temperature feedback recursion of the LNN remained relatively stable under the ideal condition in which future exogenous variables were accurately available. In contrast, strict recursive closed-loop forecasting no longer used observed future exogenous variables and instead relied on the exogenous-variable generator to construct future inputs. Its MAE/RMSE values increased to 0.6607/0.8392 °C, 0.6925/0.8753 °C, and 0.8071/1.0341 °C at the 6 h, 24 h, and 48 h horizons, respectively. These errors increased with the forecasting horizon, showing that exogenous-variable generation errors further amplified long-horizon closed-loop prediction errors after entering the input window.

The persistence baseline for exogenous variables produced substantially higher errors than the other two protocols. Its MAE/RMSE values were 1.2172/1.5803 °C, 2.1037/2.6012 °C, and 2.0525/2.5313 °C at the 6 h, 24 h, and 48 h horizons, respectively. These results show that simply keeping future exogenous variables equal to the most recent observations at the forecast origin could not represent the diurnal cycles and short-term fluctuations of environmental driving variables such as solar radiation, outdoor temperature and humidity, and wind speed. Figure 11 shows the error differences among the three protocols. Semi-closed-loop forecasting had the lowest errors, strict recursive closed-loop forecasting had higher errors than semi-closed-loop forecasting but much lower errors than the persistence baseline, and the persistence baseline showed substantial error increases at the 24 h and 48 h horizons. These results indicate that although the exogenous-variable generator introduced generation errors, it still provided more effective future driving information than the simple persistence strategy.

Quantitatively, compared with semi-closed-loop forecasting, strict recursive closed-loop forecasting increased MAE and RMSE by 29.70% and 30.68% at 6 h, 35.68% and 36.23% at 24 h, and 58.04% and 61.05% at 48 h, respectively. This progressively widening difference indicates that the use of observed future exogenous variables increasingly underestimated deployment-oriented forecasting errors as the prediction horizon extended. Compared with the persistence baseline, strict recursive closed-loop forecasting reduced MAE and RMSE by 45.72% and 46.90% at 6 h, 67.08% and 66.35% at 24 h, and 60.68% and 59.15% at 48 h, respectively. From 6 h to 48 h, the MAE and RMSE under semi-closed-loop forecasting remained nearly unchanged, with changes of 0.26% and −0.02%, whereas they increased by 22.16% and 23.22% under strict recursive closed-loop forecasting and by 68.62% and 60.18% under the persistence baseline. These results quantitatively demonstrate both the underestimation caused by ideal future exogenous inputs and the benefit of generated exogenous variables relative to a simple persistence assumption.

Overall, using observed future exogenous variables clearly underestimated the prediction errors of the model under practical online deployment conditions, whereas strict recursive closed-loop forecasting more realistically reflected the recursive process jointly affected by predicted-temperature feedback and exogenous-variable generation errors. The ablation experiment demonstrated that multi-horizon greenhouse air temperature forecasting should not rely only on ideal exogenous-driving conditions or semi-closed-loop evaluation. Instead, long-term model stability should be tested under strict recursive closed-loop conditions. For the LNN, the results showed relatively stable closed-loop recursion when observed future exogenous variables were available, but its performance under strict recursive closed-loop conditions was still constrained by future exogenous-variable generation errors. Therefore, subsequent model optimization should focus on improving exogenous-variable generation accuracy and suppressing closed-loop error accumulation.

## 4. Discussion

### 4.1. Significance of Strict Recursive Closed-Loop Evaluation for Greenhouse Air Temperature Forecasting

Greenhouse air temperature forecasting models are ultimately intended to support online monitoring, low-temperature warning, ventilation regulation, auxiliary heating, and model predictive control. Their key requirement is not only to achieve low single-step errors on offline test sets, but also to generate reliable temperature trajectories over future control windows based on historical sensor observations. Previous studies have shown that model predictive control can coordinate greenhouse environmental targets and energy consumption, and data-driven forecasting models are becoming important components of greenhouse temperature control and energy-use assessment [3,4,33]. From the perspective of sensor-system applications, a deployed forecasting model can only access observations collected before the forecast origin, whereas future greenhouse air temperature and future exogenous sensor variables are unavailable. Software sensor and soft-measurement studies have also shown that computational models based on hardware sensing data are useful for estimating variables that are difficult to measure directly or obtain in real time [17,18]. Therefore, if observed future exogenous variables are used during evaluation, the resulting errors may represent an ideal information condition rather than realistic online deployment performance.

From a control perspective, these forecasting differences may directly affect the timing and intensity of greenhouse regulation actions. If a controller is designed using errors obtained under semi-closed-loop evaluation, its expected performance may be overly optimistic because future exogenous disturbances are assumed to be known. In practical model predictive control, underestimated forecasting uncertainty may lead to delayed heating or ventilation, insufficient shading before rapid daytime warming, or unnecessary energy consumption caused by overly aggressive corrective actions. Therefore, prediction models intended for greenhouse control should be evaluated under the same information constraints encountered during deployment, and the resulting forecast uncertainty should be incorporated into control constraints, safety margins, or robust decision rules rather than relying only on point predictions.

The ablation results in this study confirmed this effect. When observed future exogenous variables were used, the MAE of the LNN remained approximately 0.5094–0.5107 °C across the 6 h, 24 h, and 48 h horizons. Under strict recursive closed-loop conditions, future exogenous variables were recursively generated by the generator, and the MAE increased to 0.6607, 0.6925, and 0.8071 °C, respectively. This difference indicates that using observed future exogenous sensor variables can substantially underestimate long-horizon deployment errors and may lead to overestimation of subsequent control performance and energy-saving potential. This finding is consistent with multi-step time-series forecasting studies showing that recursive prediction is prone to error propagation [19], and with Scheduled Sampling research indicating that the mismatch between observed inputs during training and model-generated inputs during inference can induce error accumulation [20]. In greenhouse air temperature forecasting, this issue is further amplified because errors arise not only from predicted-temperature feedback, but also from uncertainty in future exogenous sensor variables. Therefore, strict recursive closed-loop evaluation is necessary for shifting greenhouse forecasting assessment from offline fitting accuracy toward online deployability.

### 4.2. Exogenous-Variable Generation Error as an Important Constraint on Long-Horizon Forecasting

Greenhouse air temperature is jointly affected by outdoor meteorological conditions, solar radiation, heat transfer through covering structures, ventilation heat exchange, heat storage and release by soil and structural components, and crop transpiration [1,2]. In short-horizon forecasting, the model can rely more heavily on recent historical temperature and environmental states for extrapolation. However, when the forecasting horizon is extended to 24 h or 48 h, the reliability of future driving variables becomes a key factor affecting the stability of predicted temperature trajectories. The results of this study showed that exogenous-variable generation errors generally increased with the extension of the forecasting horizon. Outdoor temperature, solar radiation, soil moisture, and greenhouse relative humidity showed relatively pronounced error accumulation over longer horizons. In particular, the MAE and RMSE of solar radiation reached 108.09 μmol m^−2^ s^−1^ and 175.51 μmol m^−2^ s^−1^ at the 48 h horizon, respectively, indicating that the simple linear exogenous-variable generator was still limited in reconstructing radiation peaks, weather fluctuations, and shading-related changes.

The influence of exogenous-variable errors on greenhouse air temperature prediction was variable-specific and was not determined solely by the magnitude of generation errors. Error propagation analysis showed that outdoor temperature and solar radiation errors had the most stable correlations with greenhouse air temperature prediction errors, and these correlations increased as the forecasting horizon extended. The Spearman correlation coefficient between outdoor-temperature error and temperature prediction error increased from 0.3119 at 6 h to 0.4674 at 48 h, while the corresponding coefficient for solar radiation increased from 0.1928 to 0.2955. Outdoor temperature determines the heat-exchange boundary between the greenhouse and the external environment, whereas solar radiation is the main energy input for daytime warming. Therefore, biases in these two variables are more likely to cause deviations in warming rate, peak temperature, and night-time cooling trends. Recent data-driven weather forecasting studies have also emphasized that uncertainty in future meteorological drivers affects multi-horizon forecasting and downstream decision-making [20,21]. From the perspective of greenhouse microclimate forecasting, this study further indicates that future exogenous variables should not be treated simply as known inputs; instead, they should be independently modelled and evaluated as key sources of uncertainty in strict recursive closed-loop long-horizon forecasting.

### 4.3. Model Applicability, Limitations, and Future Work

The LNN was introduced as a continuous-time candidate model because greenhouse air temperature exhibits continuous evolution, multi-time-scale variation, and thermal inertia. The repeated-run results showed that the LNN achieved significantly lower one-step prediction errors, whereas LSTM achieved significantly lower MAE and RMSE values under the 6 h, 24 h, and 48 h strict recursive closed-loop forecasting horizons. This differentiated performance may be related to the error-transmission characteristics of the two recurrent structures. The continuous state updates and learnable time constants of the LNN may support rapid responses to recent environmental changes and improve short-term fitting; however, small errors in recursively reconstructed inputs may also be progressively integrated into the liquid-state trajectory. By contrast, the LSTM cell-state pathway and its input, forget, and output gates explicitly regulate information retention and replacement, which may help preserve slower greenhouse thermal trends while attenuating short-term perturbations introduced by recursive feedback [24,25]. Because hidden-state sensitivity and state drift were not directly analysed, this explanation should be regarded as a mechanism-based interpretation rather than direct causal evidence. Therefore, the LNN should be considered a theoretically relevant continuous-time candidate with strong short-term fitting ability, rather than a model proven to be superior across all forecasting horizons.

This study has several limitations. First, the linear exogenous-variable generator had limited ability to represent abrupt solar-radiation changes, random wind-speed fluctuations, irrigation-induced soil-moisture variation, and other management disturbances. Second, the study was based on one tomato solar greenhouse in Alar over a single year. Therefore, the reported performance cannot establish direct transferability to different greenhouse structures, crops, climatic regions, management regimes, sensor layouts, or sampling intervals and should be interpreted as site-specific evidence for the proposed evaluation framework. Third, the strict recursive setting used generated exogenous variables rather than archived weather-forecast products.

Future work should compare linear, nonlinear, and probabilistic exogenous-variable generators and incorporate weather forecasts, facility-operation states, and management-event records. Multi-scenario validation should cover solar greenhouses, arched plastic greenhouses, and Venlo-type glass greenhouses across different crops, seasons, and climatic zones. Site-held-out or leave-one-greenhouse-out evaluation should be used to compare direct transfer and local recalibration under the same recursive forecasting protocol, while compatible public greenhouse microclimate datasets may provide additional external validation after variable and temporal harmonization. Real-greenhouse model predictive control experiments are also needed to determine how recursive forecasting errors affect actuator timing, temperature-constraint violations, environmental stability, and energy consumption. Future controllers should further compare deterministic MPC based on point forecasts with robust or uncertainty-aware MPC strategies that explicitly account for exogenous-variable generation errors [34,35].

## 5. Conclusions

This study designed a strict recursive closed-loop forecasting protocol and an exogenous-error propagation analysis framework for greenhouse air temperature prediction based on multi-source environmental sensing data. Within this framework, an LNN model was constructed as a continuous-time forecasting model to evaluate the feasibility of recursive closed-loop prediction, and its performance was compared with those of LSTM, GRU, and XGBoost under the same data partitioning, input variables, historical window length, and evaluation metrics. Across 10 repeated runs, the LNN achieved the lowest one-step prediction error, with an MAE of 0.4771 °C and an RMSE of 0.5996 °C, indicating good short-term fitting ability. However, under strict recursive closed-loop forecasting over the 6 h, 24 h, and 48 h horizons, LSTM achieved the lowest MAE and RMSE values, demonstrating stronger long-horizon recursive stability. The LNN remained more accurate than GRU and XGBoost at some long horizons, but it was not the best-performing model under the strict recursive setting. These results indicate that one-step prediction accuracy and long-horizon recursive stability should be evaluated separately in greenhouse air temperature forecasting.

At the same time, this study proposed an exogenous-variable generator to generate future exogenous inputs during strict recursive closed-loop forecasting. The results showed that the exogenous-variable generator could generally maintain the variation trends of the main environmental variables, but the generation difficulty differed among variables. The outdoor temperature generation error increased from an MAE of 1.38 °C at 6 h to 1.96 °C at 48 h. The MAE and RMSE of solar radiation reached 108.09 μmol m^−2^ s^−1^ and 175.51 μmol m^−2^ s^−1^, respectively, in 48 h forecasting. These results indicate that outdoor temperature and solar radiation are the main uncertainty sources in long-horizon forecasting. The ablation experiments further showed that the semi-closed-loop setting using observed future exogenous variables significantly underestimated model errors. In 48 h forecasting, the MAE and RMSE of strict recursive closed-loop forecasting increased by 58.04% and 61.05%, respectively, compared with semi-closed-loop forecasting, but decreased by 60.68% and 59.15%, respectively, compared with the persistence baseline. These results verified the necessity of strict recursive closed-loop evaluation and the practical forecasting value of the model.

In summary, the main contribution of this study is the establishment of a strict recursive closed-loop evaluation framework for greenhouse air temperature forecasting under conditions in which future sensor observations are unavailable. The results showed that the LNN improved one-step prediction accuracy, whereas LSTM provided stronger long-horizon recursive stability. Strict recursive closed-loop evaluation more realistically reflected error accumulation during long-horizon recursion when future sensor observations were unavailable. Exogenous-error propagation analysis further identified the main effects of key sensing variables, such as outdoor temperature and solar radiation, on temperature prediction errors. These results provide a basis for the selection, evaluation, and optimization of greenhouse air temperature forecasting models and offer a reference for greenhouse environmental online prediction and control decision-making based on sensor monitoring data. In practical greenhouse management, such multi-horizon temperature trajectories can provide early information for heating, ventilation, shading, irrigation scheduling, and energy-use optimization, especially when control decisions require sufficient lead time. However, before direct application to other greenhouses, the model should be recalibrated or revalidated using local sensor data, because its performance may be affected by greenhouse geometry, covering materials, ventilation mode, sensor placement, local climate, crop conditions, and sampling interval.

## Figures and Tables

**Table 1:** Main technical specifications of the environmental sensors.

Position	Monitoring Variable	Sensor/Model	Measurement Range	Accuracy
Indoor, 1.5 m height	Air temperature	RS-WS-LORAH-2-2	−40–120 °C	±0.5 °C
	Relative humidity	RS-WS-LORAH-2-2	0–100% RH	±2% RH
	CO_2_ concentration	RS-CO_2_-LORAH-DC-2	0–5000 ppm	±40 ppm
Soil, 20 cm depth	Soil moisture	RS-TR-LORAH-2-WS	0–100%	±2%
Outdoor weather station	Air temperature	RS-BYH-M	−40–120 °C	±0.5 °C
	Relative humidity	RS-BYH-M	0–99% RH	±3% RH
	Photosynthetically active radiation (PAR)	RS-GH-N01-AL	0–2000 μmol m^−2^ s^−1^	±5%
	Wind speed	RS-FSA-4G	0–60 m s^−1^	±0.2 m s^−1^

**Table 2:** Three closed-loop forecasting protocols.

Prediction Protocol	Future Greenhouse Air Temperature	Future Exogenous Variables
Semi-closed-loop forecasting	Predicted value feedback	Observed future exogenous variables
Strict recursive closed-loop forecasting	Predicted value feedback	Generated exogenous variables
Persistence exogenous-variable baseline	Predicted value feedback	Persistence exogenous variables

**Table 3:** Repeated-run performance comparison of different models in one-step prediction and strict recursive closed-loop forecasting.

Forecast Horizon	Metric	LNN (°C)	LSTM (°C)	GRU (°C)	XGBoost (°C)
One-step	MAE	0.4771 ± 0.0009	0.5103 ± 0.0012	0.5314 ± 0.0017	0.5158 ± 0.0021
One-step	RMSE	0.5996 ± 0.0010	0.6396 ± 0.0014	0.6622 ± 0.0015	0.6457 ± 0.0018
6 h	MAE	0.6607 ± 0.0054	0.6032 ± 0.0041	0.6550 ± 0.0045	0.7080 ± 0.0006
6 h	RMSE	0.8392 ± 0.0071	0.7608 ± 0.0056	0.8170 ± 0.0061	0.9000 ± 0.0022
24 h	MAE	0.6925 ± 0.0079	0.6227 ± 0.0059	0.7271 ± 0.0092	0.7539 ± 0.0041
24 h	RMSE	0.8753 ± 0.0097	0.7846 ± 0.0079	0.9023 ± 0.0119	0.9522 ± 0.0096
48 h	MAE	0.8071 ± 0.0122	0.6794 ± 0.0097	0.8486 ± 0.0135	0.9051 ± 0.0025
48 h	RMSE	1.0341 ± 0.0147	0.8621 ± 0.0128	1.0560 ± 0.0177	1.1599 ± 0.0210

Note: Values are presented as mean ± standard deviation in °C over 10 repeated runs. Friedman test results are provided in Table A3, and complete Holm-adjusted pairwise comparisons are provided in Supplementary Table S1.

**Table 4:** Statistics of exogenous-variable generation errors under different forecasting horizons.

Forecasting Horizon	Exogenous Variable	Unit for MAE/RMSE/Bias	MAE	RMSE	Bias	NRMSE
6 h	Outdoor temperature	°C	1.38	1.81	−0.74	0.054
	Outdoor relative humidity	% RH	3.38	4.37	−1.43	0.069
	Solar radiation	μmol m^−2^ s^−1^	76.83	128.10	−25.17	0.109
	Wind speed	m s^−1^	0.85	1.03	0.01	0.168
	Soil moisture	%	3.05	4.84	0.57	0.084
	CO_2_ concentration	ppm	9.02	11.57	0.14	0.111
	Indoor relative humidity	% RH	3.75	5.01	−1.15	0.101
	VPD	kPa	0.11	0.14	0.01	0.092
24 h	Outdoor temperature	°C	1.52	1.92	−0.83	0.057
	Outdoor relative humidity	% RH	3.73	4.69	−1.55	0.074
	Solar radiation	μmol m^−2^ s^−1^	82.69	134.60	−28.15	0.115
	Wind speed	m s^−1^	0.85	1.03	0.01	0.169
	Soil moisture	%	6.35	9.50	2.10	0.165
	CO_2_ concentration	ppm	9.72	12.30	0.24	0.118
	Indoor relative humidity	% RH	4.17	5.50	−1.66	0.111
	VPD	kPa	0.12	0.15	0.02	0.098
48 h	Outdoor temperature	°C	1.96	2.53	−1.18	0.075
	Outdoor relative humidity	% RH	4.65	5.90	−2.18	0.093
	Solar radiation	μmol m^−2^ s^−1^	108.09	175.51	−35.21	0.149
	Wind speed	m s^−1^	0.85	1.03	0.01	0.169
	Soil moisture	%	7.07	10.33	2.48	0.180
	CO_2_ concentration	ppm	10.19	12.86	0.43	0.124
	Indoor relative humidity	% RH	4.69	6.24	−2.28	0.125
	VPD	kPa	0.13	0.17	0.03	0.108

Note: MAE, RMSE, and Bias are expressed in the unit listed for each exogenous variable and are rounded to two decimal places. NRMSE is dimensionless and is rounded to three decimal places. VPD is a derived variable reconstructed from temperature and humidity.

**Table 5:** Comparison of LNN prediction errors under different closed-loop forecasting protocols.

Forecasting Protocol	Future Greenhouse Air Temperature	Future Exogenous Variables	6 h MAE/RMSE (°C)	24 h MAE/RMSE (°C)	48 h MAE/RMSE (°C)
Semi-closed-loop forecasting	Predicted value feedback	Observed exogenous variables	0.5094/0.6422	0.5104/0.6425	0.5107/0.6421
Strict recursive closed-loop forecasting	Predicted value feedback	Generated exogenous variables	0.6607/0.8392	0.6925/0.8753	0.8071/1.0341
Persistence exogenous-variable baseline	Predicted value feedback	Persistence exogenous variables	1.2172/1.5803	2.1037/2.6012	2.0525/2.5313

Note: MAE and RMSE are expressed in °C.

## Data Availability

The processed data, random-seed settings, complete repeated-run results, and statistical-analysis results used in this study are available from the corresponding authors upon reasonable request. The run-level performance results and complete Holm-adjusted pairwise comparisons are provided in the Supplementary Materials.

## Supplementary Materials

The following supporting information can be downloaded at: https://www.mdpi.com/article/10.3390/s26154717/s1, Figure S1: Diagnostic comparison of the linear and Residual-GBRT exogenous-variable generators; Table S1: Pairwise Wilcoxon signed-rank tests with Holm correction for repeated model performance; Table S2: Spearman correlations between absolute exogenous-variable generation errors and absolute greenhouse air temperature prediction errors under different forecasting horizons; Table S3: Comparison of linear and Residual-GBRT exogenous-variable generators under the 6 h, 24 h, and 48 h recursive forecasting horizons; Table S4: Validation-based one-factor-at-a-time hyperparameter sensitivity analysis for LNN, LSTM, GRU, and the tree-boosting baseline.

## Abbreviations

The following abbreviations are used in this manuscript:

ANN	Artificial neural network
LCs	Liquid cells
LNN	Liquid Neural Network
GRU	Gated recurrent unit
XGBoost	Extreme gradient boosting
LSTM	Long short-term memory
IoT	Internet of Things
RMSE	Root mean square error
MAE	Mean absolute error
PAR	Photosynthetically active radiation
RH	Relative humidity
NRMSE	Normalized root mean square error
RNN	Recurrent neural network
VPD	Vapor pressure deficit

## Appendix A

**Table A1:** One-factor sensitivity analysis of key LNN hyperparameters under recursive forecasting horizons.

Analysis Item	Setting	MAE 6 h	RMSE 6 h	MAE 24 h	RMSE 24 h	MAE 48 h	RMSE 48 h
Liquid neurons	32	0.6899	0.8706	0.6958	0.8771	0.8041	1.0238
Liquid neurons	64	0.6841	0.8632	0.6911	0.8709	0.7998	1.0193
Liquid neurons	128	0.6891	0.8707	0.6964	0.8786	0.8071	1.0294
Batch size	16	0.6854	0.8657	0.6927	0.8738	0.8002	1.0187
Batch size	32	0.6841	0.8632	0.6911	0.8709	0.7998	1.0193
Batch size	64	0.6898	0.8698	0.6967	0.8771	0.8071	1.0254
Gradient clipping threshold	0.5	0.6778	0.8549	0.6856	0.8639	0.7924	1.0101
Gradient clipping threshold	1.0	0.6841	0.8632	0.6911	0.8709	0.7998	1.0193
Gradient clipping threshold	2.0	0.6852	0.8644	0.6921	0.8721	0.8011	1.0206
Learning-rate schedule	Without warmup	0.6791	0.8564	0.6854	0.8636	0.7924	1.0096
Learning-rate schedule	Warmup + cosine annealing	0.6841	0.8632	0.6911	0.8709	0.7998	1.0193

Note: MAE and RMSE are expressed in °C. The 6 h, 24 h, and 48 h horizons correspond to 12, 48, and 96 recursive prediction steps, respectively, because the sensor sampling interval was 30 min. In each one-factor sensitivity analysis, only the listed hyperparameter was changed, while the remaining settings were kept unchanged.

**Table A2:** Final model architectures and training settings used in this study.

Setting Item	LNN	LSTM	GRU	Tree-Boosting Baseline
Input setting	39 features; L = 36	39 features; L = 36	39 features; L = 36	39 features; L = 36, flattened to 1404 predictors
Architecture	64 liquid neurons; continuous recurrent liquid state	2 LSTM layers; 64 hidden units; dropout = 0.3	2 GRU layers; 64 hidden units; dropout = 0.3	Gradient-boosted regression trees implemented using XGBoost
Optimizer/learner	Custom AdamW	Adam	Adam	XGBoost regressor
Learning rate	1 × 10^−3^ max; 1 × 10^−5^ min	8 × 10^−4^	1 × 10^−3^	0.05
Batch size	32	32	32	—
Epochs/rounds	200	80	80	120
Loss function	Huber loss (δ = 0.5)	MSE	MSE	Squared-error objective
Validation/model selection	Lowest validation Huber loss; patience = 35	Best validation-loss network	Best validation-loss network	Best learner count based on validation cumulative loss
Other settings	Weight decay = 1 × 10^−4^; gradient clipping = 1.0; 10-epoch warmup + cosine annealing	Piecewise learning-rate schedule; drop factor = 0.5	No learning-rate schedule	MaxNumSplits = 16; MinLeafSize = 5

**Table A3:** Friedman test results for repeated model performance over 10 random-seed runs.

Forecast Horizon	Metric	*n*	Friedman χ^2^	df	*p*-Value
One-step	MAE	10	30.00	3	1.38 × 10^−6^
One-step	RMSE	10	30.00	3	1.38 × 10^−6^
6 h	MAE	10	28.08	3	3.49 × 10^−6^
6 h	RMSE	10	30.00	3	1.38 × 10^−6^
24 h	MAE	10	30.00	3	1.38 × 10^−6^
24 h	RMSE	10	28.08	3	3.49 × 10^−6^
48 h	MAE	10	30.00	3	1.38 × 10^−6^
48 h	RMSE	10	28.08	3	3.49 × 10^−6^
