# Supplementary material for: Strict Recursive Closed-Loop Evaluation of Greenhouse Air Temperature Forecasting with Generated Exogenous Variables and Exogenous-Error Propagation Analysis
Source: Sensors (Basel). 2026 Jul 24;26(15):4717. doi: 10.3390/s26154717 (PMC13468724; doi:10.3390/s26154717)
Supplement: Supplementary file 1 [file sensors-26-04717-s001.zip › sensors-4428542-supplementary.pdf]

Supplementary Table S1. Pairwise Wilcoxon signed-rank tests with Holm correction for repeated model performance.

Forecast horizon	Metric	Comparison	Mean difference (A − B)	W	Raw p-value	Holm-adjusted p-value	Significant
One-step	MAE	LNN vs LSTM	-0.0332	0	0.00195	0.0117	Yes
One-step	MAE	LNN vs GRU	-0.0543	0	0.00195	0.0117	Yes
One-step	MAE	LNN vs XGBoost	-0.0387	0	0.00195	0.0117	Yes
One-step	MAE	LSTM vs GRU	-0.0211	0	0.00195	0.0117	Yes
One-step	MAE	LSTM vs XGBoost	-0.0055	0	0.00195	0.0117	Yes
One-step	MAE	GRU vs XGBoost	0.0156	0	0.00195	0.0117	Yes
One-step	RMSE	LNN vs LSTM	-0.0400	0	0.00195	0.0117	Yes
One-step	RMSE	LNN vs GRU	-0.0626	0	0.00195	0.0117	Yes
One-step	RMSE	LNN vs XGBoost	-0.0461	0	0.00195	0.0117	Yes
One-step	RMSE	LSTM vs GRU	-0.0226	0	0.00195	0.0117	Yes
One-step	RMSE	LSTM vs XGBoost	-0.0061	0	0.00195	0.0117	Yes
One-step	RMSE	GRU vs XGBoost	0.0165	0	0.00195	0.0117	Yes
6 h	MAE	LNN vs LSTM	0.0574	0	0.00195	0.0117	Yes
6 h	MAE	LNN vs GRU	0.0056	9	0.0645	0.0645	No
6 h	MAE	LNN vs XGBoost	-0.0474	0	0.00195	0.0117	Yes
6 h	MAE	LSTM vs GRU	-0.0518	0	0.00195	0.0117	Yes
6 h	MAE	LSTM vs XGBoost	-0.1048	0	0.00195	0.0117	Yes
6 h	MAE	GRU vs XGBoost	-0.0530	0	0.00195	0.0117	Yes
6 h	RMSE	LNN vs LSTM	0.0784	0	0.00195	0.0117	Yes
6 h	RMSE	LNN vs GRU	0.0222	0	0.00195	0.0117	Yes
6 h	RMSE	LNN vs XGBoost	-0.0608	0	0.00195	0.0117	Yes
6 h	RMSE	LSTM vs GRU	-0.0562	0	0.00195	0.0117	Yes
6 h	RMSE	LSTM vs XGBoost	-0.1392	0	0.00195	0.0117	Yes
6 h	RMSE	GRU vs XGBoost	-0.0830	0	0.00195	0.0117	Yes

Forecast horizon	Metric	Comparison	Mean difference (A − B)	W	Raw p-value	Holm-adjusted p-value	Significant
24 h	MAE	LNN vs LSTM	0.0698	0	0.00195	0.0117	Yes
24 h	MAE	LNN vs GRU	-0.0346	0	0.00195	0.0117	Yes
24 h	MAE	LNN vs XGBoost	-0.0614	0	0.00195	0.0117	Yes
24 h	MAE	LSTM vs GRU	-0.1044	0	0.00195	0.0117	Yes
24 h	MAE	LSTM vs XGBoost	-0.1312	0	0.00195	0.0117	Yes
24 h	MAE	GRU vs XGBoost	-0.0268	0	0.00195	0.0117	Yes
24 h	RMSE	LNN vs LSTM	0.0907	0	0.00195	0.0117	Yes
24 h	RMSE	LNN vs GRU	-0.0270	3	0.00977	0.0117	Yes
24 h	RMSE	LNN vs XGBoost	-0.0769	0	0.00195	0.0117	Yes
24 h	RMSE	LSTM vs GRU	-0.1177	0	0.00195	0.0117	Yes
24 h	RMSE	LSTM vs XGBoost	-0.1676	0	0.00195	0.0117	Yes
24 h	RMSE	GRU vs XGBoost	-0.0499	0	0.00195	0.0117	Yes
48 h	MAE	LNN vs LSTM	0.1277	0	0.00195	0.0117	Yes
48 h	MAE	LNN vs GRU	-0.0415	0	0.00195	0.0117	Yes
48 h	MAE	LNN vs XGBoost	-0.0980	0	0.00195	0.0117	Yes
48 h	MAE	LSTM vs GRU	-0.1692	0	0.00195	0.0117	Yes
48 h	MAE	LSTM vs XGBoost	-0.2257	0	0.00195	0.0117	Yes
48 h	MAE	GRU vs XGBoost	-0.0565	0	0.00195	0.0117	Yes
48 h	RMSE	LNN vs LSTM	0.1720	0	0.00195	0.0117	Yes
48 h	RMSE	LNN vs GRU	-0.0219	7	0.0371	0.0371	Yes
48 h	RMSE	LNN vs XGBoost	-0.1258	0	0.00195	0.0117	Yes
48 h	RMSE	LSTM vs GRU	-0.1939	0	0.00195	0.0117	Yes
48 h	RMSE	LSTM vs XGBoost	-0.2978	0	0.00195	0.0117	Yes
48 h	RMSE	GRU vs XGBoost	-0.1039	0	0.00195	0.0117	Yes

Note: All tests were based on 10 paired runs using fixed random seeds 42, 123, 456, 789, 1024, 2024, 2025, 2026, 2048, and 3407. Pairwise comparisons were conducted only after a significant Friedman test. Holm correction was applied to the six pairwise model comparisons within each forecasting-horizon–metric

combination. Mean difference was calculated as Model A minus Model B; therefore, a negative value indicates a lower prediction error for Model A. W denotes the Wilcoxon signed-rank statistic. Holm-adjusted p-values below 0.05 were considered statistically significant.

Supplementary Table S2. Spearman correlations between absolute exogenous-variable generation errors and absolute greenhouse air temperature prediction errors under different forecasting horizons.

Forecast horizon	Exogenous variable	N	Spearman ρ	Raw p-value	BH-FDR-adjusted p-value	Significance
6 h	Outdoor temperature	31,416	0.3119	$<1.0 \times 10^{-300}$	$<1.0 \times 10^{-300}$	***
	Outdoor relative humidity	31,416	0.0024	0.6769	0.6769	ns
	Solar radiation	31,416	0.1928	7.93×10^{-261}	2.11×10^{-260}	***
	Wind speed	31,416	0.0223	7.58×10^{-5}	8.66×10^{-5}	***
	Soil moisture	31,416	0.0752	1.32×10^{-40}	2.43×10^{-40}	***
	CO_2 concentration	31,416	0.0545	4.09×10^{-22}	6.13×10^{-22}	***
	Indoor relative humidity	31,416	0.0101	0.0725	0.0757	ns
	VPD	31,416	0.0979	1.13×10^{-67}	2.26×10^{-67}	***
24 h	Outdoor temperature	123,936	0.3294	$<1.0 \times 10^{-300}$	$<1.0 \times 10^{-300}$	***
	Outdoor relative humidity	123,936	−0.0631	1.58×10^{-109}	3.45×10^{-109}	***
	Solar radiation	123,936	0.2068	$<1.0 \times 10^{-300}$	$<1.0 \times 10^{-300}$	***
	Wind speed	123,936	0.0160	1.83×10^{-8}	2.31×10^{-8}	***
	Soil moisture	123,936	0.0335	3.59×10^{-32}	5.75×10^{-32}	***
	CO_2 concentration	123,936	0.0360	8.65×10^{-37}	1.48×10^{-36}	***
	Indoor relative humidity	123,936	−0.0135	1.96×10^{-6}	2.36×10^{-6}	***
	VPD	123,936	0.0764	1.43×10^{-159}	3.43×10^{-159}	***
48 h	Outdoor temperature	243,264	0.4674	$<1.0 \times 10^{-300}$	$<1.0 \times 10^{-300}$	***
	Outdoor relative humidity	243,264	−0.1009	$<1.0 \times 10^{-300}$	$<1.0 \times 10^{-300}$	***
	Solar radiation	243,264	0.2955	$<1.0 \times 10^{-300}$	$<1.0 \times 10^{-300}$	***
	Wind speed	243,264	0.0172	2.52×10^{-17}	3.56×10^{-17}	***
	Soil moisture	243,264	0.0069	6.77×10^{-4}	7.38×10^{-4}	***
	CO_2 concentration	243,264	0.0800	$<1.0 \times 10^{-300}$	$<1.0 \times 10^{-300}$	***
	Indoor relative humidity	243,264	−0.0121	2.08×10^{-9}	2.78×10^{-9}	***

Forecast horizon	Exogenous variable	N	Spearman ρ	Raw p-value	BH-FDR-adjusted p-value	Significance
	VPD	243,264	0.0935	$<1.0 \times 10^{-300}$	$<1.0 \times 10^{-300}$	***

Note: Spearman correlation coefficients were calculated between the absolute generation error of each exogenous variable and the absolute greenhouse air temperature prediction error. All significance tests were two-sided. The 24 raw p-values obtained for eight exogenous variables across the 6 h, 24 h, and 48 h forecasting horizons were adjusted using the Benjamini–Hochberg false discovery rate procedure. ***, **, and * indicate FDR-adjusted $p<0.001$, $p<0.01$, and $p<0.05$, respectively; ns indicates $p\geq0.05$. N denotes the number of paired error observations used in each correlation analysis. VPD denotes vapor pressure deficit.

Supplementary Table S3. Comparison of linear and Residual-GBRT exogenous-variable generators under the 6 h, 24 h, and 48 h recursive forecasting horizons.

Panel A. Pooled generation-error metrics and relative changes.

Horizon	Exogenous variable	Linear MAE	Linear RMSE	Linear Bias	Linear NRMSE	Residual-GBRT MAE	Residual-GBRT RMSE	Residual-GBRT Bias	Residual-GBRT NRMSE	MAE change (%)	RMSE change (%)	NRMSE change (%)
6 h	Outdoor temperature	1.3813	1.8074	-0.7445	0.0536	1.4205	1.8838	-1.0217	0.0558	-2.84	-4.23	-4.23
6 h	Outdoor relative humidity	3.3768	4.3705	-1.4336	0.0688	3.0238	3.9216	-1.0596	0.0617	10.45	10.27	10.27
6 h	Photosynthetically active radiation	76.8295	128.0967	-25.1739	0.1094	66.1469	116.6684	-38.8039	0.0997	13.90	8.92	8.92
6 h	Wind speed	0.8523	1.0267	0.0054	0.1680	0.8205	0.9862	-0.0028	0.1614	3.73	3.94	3.94
6 h	Soil moisture	3.0485	4.8352	0.5700	0.0841	3.0719	5.2188	1.0061	0.0908	-0.77	-7.93	-7.93
6 h	CO_2 concentration	9.0171	11.5672	0.1399	0.1113	8.1534	10.5039	0.2443	0.1011	9.58	9.19	9.19
6 h	Indoor relative humidity	3.7514	5.0121	-1.1458	0.1007	3.3429	4.4360	-0.8713	0.0891	10.89	11.49	11.49
24 h	Outdoor temperature	1.5220	1.9236	-0.8323	0.0570	1.5719	2.0160	-1.1941	0.0598	-3.28	-4.80	-4.80
24 h	Outdoor relative humidity	3.7279	4.6942	-1.5529	0.0739	3.2643	4.1253	-1.2948	0.0649	12.44	12.12	12.12
24 h	Photosynthetically active radiation	82.6826	134.5990	-28.1483	0.1150	69.5511	120.2754	-39.6328	0.1027	15.88	10.64	10.64
24 h	Wind speed	0.8547	1.0295	0.0022	0.1685	0.8206	0.9855	0.0176	0.1613	3.99	4.28	4.28
24 h	Soil moisture	6.3466	9.5084	2.0969	0.1654	6.7924	10.8435	4.3133	0.1886	-7.03	-14.04	-14.04
24 h	CO_2 concentration	9.7207	12.2918	0.2374	0.1183	8.7843	11.1689	0.3369	0.1075	9.63	9.14	9.14

Horizon	Exogenous variable	Linear MAE	Linear RMSE	Linear Bias	Linear NRMSE	Residual-GBRT MAE	Residual-GBRT RMSE	Residual-GBRT Bias	Residual-GBRT NRMSE	MAE change (%)	RMSE change (%)	NRMSE change (%)
24 h	Indoor relative humidity	4.1675	5.5070	-1.6601	0.1107	3.5787	4.7168	-1.1465	0.0948	14.13	14.35	14.35
48 h	Outdoor temperature	1.9552	2.5348	-1.1837	0.0751	1.9804	2.5619	-1.6423	0.0759	-1.29	-1.07	-1.07
48 h	Outdoor relative humidity	4.6488	5.8975	-2.1758	0.0928	3.9205	4.9430	-1.8256	0.0778	15.67	16.18	16.18
48 h	Photosynthetically active radiation	108.0877	175.5103	-35.2133	0.1493	83.7269	145.7563	-50.9838	0.1240	22.54	16.95	16.95
48 h	Wind speed	0.8543	1.0319	0.0074	0.1689	0.8219	0.9871	0.0402	0.1616	3.79	4.34	4.34
48 h	Soil moisture	7.0672	10.3301	2.4803	0.1796	7.6134	11.7320	5.3811	0.2040	-7.73	-13.57	-13.57
48 h	CO_2 concentration	10.1901	12.8587	0.4327	0.1237	9.1785	11.7039	0.6466	0.1126	9.93	8.98	8.98
48 h	Indoor relative humidity	4.6857	6.2364	-2.2787	0.1253	3.8811	5.1445	-1.4527	0.1034	17.17	17.51	17.51

Panel B. Paired Wilcoxon signed-rank tests with Holm correction.

Horizon	Exogenous variable	Metric	Paired windows	Linear mean window metric	Residual-GBRT mean window metric	W	Raw p-value	Holm-adjusted p-value	Interpretation
6 h	Outdoor temperature	MAE	2618	1.3813	1.4205	1608407	0.0063	0.0266	Deterioration
6 h	Outdoor temperature	RMSE	2618	1.6184	1.6622	1590076	0.0013	0.0080	Deterioration
6 h	Outdoor relative humidity	MAE	2618	3.3768	3.0238	2681904	3.73E−138	7.45E−137	Improvement
6 h	Outdoor relative humidity	RMSE	2618	3.9686	3.5863	2682330	2.83E−138	5.94E−137	Improvement
6 h	Photosynthetically active radiation	MAE	2618	76.8295	66.1469	1227182	1.81E−112	3.27E−111	Improvement
6 h	Photosynthetically active radiation	RMSE	2618	95.9728	82.8958	1249328	4.34E−123	8.24E−122	Improvement
6 h	Wind speed	MAE	2618	0.8523	0.8205	2550238	1.28E−103	2.17E−102	Improvement
6 h	Wind speed	RMSE	2618	1.0043	0.9645	2720609	2.91E−149	6.40E−148	Improvement
6 h	Soil moisture	MAE	2618	3.0485	3.0719	1665766	0.2111	0.2111	Not significant
6 h	Soil moisture	RMSE	2618	3.6612	3.7291	1650996	0.1026	0.2052	Not significant
6 h	CO_2 concentration	MAE	2618	9.0171	8.1534	2499414	1.24E−91	1.73E−90	Improvement
6 h	CO_2 concentration	RMSE	2618	10.7609	9.7774	2542051	1.22E−101	1.96E−100	Improvement
6 h	Indoor relative humidity	MAE	2618	3.7514	3.3429	2452156	3.70E−81	4.44E−80	Improvement
6 h	Indoor relative humidity	RMSE	2618	4.4194	3.9789	2460335	6.33E−83	8.23E−82	Improvement
24 h	Outdoor temperature	MAE	2582	1.5220	1.5719	1320247	5.13E−20	4.10E−19	Deterioration
24 h	Outdoor temperature	RMSE	2582	1.8839	1.9588	1076595	8.17E−55	8.98E−54	Deterioration
24 h	Outdoor relative humidity	MAE	2582	3.7279	3.2643	3252438	<1.0E−300	<1.0E−300	Improvement

Horizon	Exogenous variable	Metric	Paired windows	Linear mean window metric	Residual-GBRT mean window metric	W	Raw p-value	Holm-adjusted p-value	Interpretation
24 h	Outdoor relative humidity	RMSE	2582	4.6150	4.0395	3275713	<1.0E−300	<1.0E−300	Improvement
24 h	Photosynthetically active radiation	MAE	2582	82.6826	69.5511	3334014	<1.0E−300	<1.0E−300	Improvement
24 h	Photosynthetically active radiation	RMSE	2582	131.0381	118.2743	3314377	<1.0E−300	<1.0E−300	Improvement
24 h	Wind speed	MAE	2582	0.8547	0.8206	3150987	<1.0E−300	<1.0E−300	Improvement
24 h	Wind speed	RMSE	2582	1.0256	0.9820	3241145	<1.0E−300	<1.0E−300	Improvement
24 h	Soil moisture	MAE	2582	6.3466	6.7924	1353501	1.20E−16	8.37E−16	Deterioration
24 h	Soil moisture	RMSE	2582	7.4805	8.1061	1222285	7.31E−32	6.58E−31	Deterioration
24 h	CO_2 concentration	MAE	2582	9.7207	8.7843	2901187	1.15E−232	2.76E−231	Improvement
24 h	CO_2 concentration	RMSE	2582	12.0319	10.9771	2891443	4.87E−229	1.12E−227	Improvement
24 h	Indoor relative humidity	MAE	2582	4.1675	3.5787	3182672	<1.0E−300	<1.0E−300	Improvement
24 h	Indoor relative humidity	RMSE	2582	5.2309	4.5279	3125116	<1.0E−300	<1.0E−300	Improvement
48 h	Outdoor temperature	MAE	2534	1.9552	1.9804	1503284	0.0053	0.0266	Deterioration
48 h	Outdoor temperature	RMSE	2534	2.4904	2.4829	1681504	0.0402	0.1205	Not significant
48 h	Outdoor relative humidity	MAE	2534	4.6488	3.9205	3193461	<1.0E−300	<1.0E−300	Improvement
48 h	Outdoor relative humidity	RMSE	2534	5.8308	4.8661	3208711	<1.0E−300	<1.0E−300	Improvement
48 h	Photosynthetically active radiation	MAE	2534	108.0877	83.7269	3211833	<1.0E−300	<1.0E−300	Improvement
48 h	Photosynthetically active radiation	RMSE	2534	167.9518	141.9219	3211777	<1.0E−300	<1.0E−300	Improvement
48 h	Wind speed	MAE	2534	0.8543	0.8219	3191533	<1.0E−300	<1.0E−300	Improvement

Horizon	Exogenous variable	Metric	Paired windows	Linear mean window metric	Residual-GBRT mean window metric	W	Raw p-value	Holm-adjusted p-value	Interpretation
48 h	Wind speed	RMSE	2534	1.0299	0.9853	3204995	<1.0E−300	<1.0E−300	Improvement
48 h	Soil moisture	MAE	2534	7.0672	7.6134	1059922	1.04E−49	1.04E−48	Deterioration
48 h	Soil moisture	RMSE	2534	8.5265	9.3816	851201	2.65E−93	3.98E−92	Deterioration
48 h	CO_2 concentration	MAE	2534	10.1901	9.1785	2844343	8.07E−248	2.10E−246	Improvement
48 h	CO_2 concentration	RMSE	2534	12.6650	11.5829	2826237	1.09E−240	2.73E−239	Improvement
48 h	Indoor relative humidity	MAE	2534	4.6857	3.8811	3144901	<1.0E−300	<1.0E−300	Improvement
48 h	Indoor relative humidity	RMSE	2534	5.9196	4.9313	3142899	<1.0E−300	<1.0E−300	Improvement

Supplementary Table S4. Validation-based one-factor-at-a-time hyperparameter sensitivity analysis for LNN, LSTM, GRU, and the tree-boosting baseline.

**Hyperparameter reference**	**Setting**	**N**	**6 h MAE**	**6 h RMSE**	**24 h MAE**	**24 h RMSE**	**48 h MAE**	**48 h RMSE**
Adopted reference configuration	64 liquid neurons; batch size 32; gradient clipping threshold 1.0; learning rate 1 × 10^{-3}; 10-epoch warmup + cosine annealing	3	0.6841 ± 0.0051	0.8632 ± 0.0067	0.6911 ± 0.0069	0.8709 ± 0.0088	0.7998 ± 0.0114	1.0193 ± 0.0147
Liquid neurons (reference = 64)	32	3	0.6899 ± 0.0062	0.8706 ± 0.0079	0.6958 ± 0.0081	0.8771 ± 0.0105	0.8041 ± 0.0132	1.0238 ± 0.0168
Liquid neurons (reference = 64)	128	3	0.6891 ± 0.0065	0.8707 ± 0.0083	0.6964 ± 0.0087	0.8786 ± 0.0112	0.8071 ± 0.0141	1.0294 ± 0.0180
Batch size (reference = 32)	16	3	0.6854 ± 0.0056	0.8657 ± 0.0071	0.6927 ± 0.0074	0.8738 ± 0.0094	0.8002 ± 0.0120	1.0187 ± 0.0155

**Hyperparameter reference**	**Setting**	**N**	**6 h MAE**	**6 h RMSE**	**24 h MAE**	**24 h RMSE**	**48 h MAE**	**48 h RMSE**
Batch size (reference = 32)	64	3	0.6898 ± 0.0064	0.8698 ± 0.0080	0.6967 ± 0.0088	0.8771 ± 0.0111	0.8071 ± 0.0145	1.0254 ± 0.0177
Gradient clipping threshold (reference = 1.0)	0.5	3	0.6778 ± 0.0059	0.8549 ± 0.0076	0.6856 ± 0.0080	0.8639 ± 0.0101	0.7924 ± 0.0126	1.0101 ± 0.0158
Gradient clipping threshold (reference = 1.0)	2.0	3	0.6852 ± 0.0055	0.8644 ± 0.0070	0.6921 ± 0.0075	0.8721 ± 0.0095	0.8011 ± 0.0122	1.0206 ± 0.0156
Learning-rate schedule	10-epoch warmup + cosine annealing (adopted)	3	0.6841 ± 0.0051	0.8632 ± 0.0067	0.6911 ± 0.0069	0.8709 ± 0.0088	0.7998 ± 0.0114	1.0193 ± 0.0147
	Without warmup	3	0.6791 ± 0.0053	0.8564 ± 0.0069	0.6854 ± 0.0071	0.8636 ± 0.0091	0.7924 ± 0.0119	1.0096 ± 0.0150
Hyperparameter reference	Setting	N	6 h MAE	6 h RMSE	24 h MAE	24 h RMSE	48 h MAE	48 h RMSE
Adopted reference configuration	64 hidden units; 2 layers; dropout 0.3; learning rate 8 × 10^{-4}	3	0.6059 ± 0.0040	0.7646 ± 0.0056	0.6229 ± 0.0060	0.7848 ± 0.0078	0.6815 ± 0.0097	0.8651 ± 0.0130
Hidden units (reference = 64)	32	3	0.6135 ± 0.0037	0.7742 ± 0.0050	0.6336 ± 0.0053	0.7982 ± 0.0072	0.6962 ± 0.0086	0.8838 ± 0.0115
Hidden units (reference = 64)	128	3	0.6051 ± 0.0052	0.7635 ± 0.0073	0.6217 ± 0.0074	0.7833 ± 0.0101	0.6899 ± 0.0126	0.8930 ± 0.0161
Layers (reference = 2)	1	3	0.6169 ± 0.0037	0.7785 ± 0.0049	0.6383 ± 0.0052	0.8042 ± 0.0071	0.7028 ± 0.0084	0.8921 ± 0.0113
Layers (reference = 2)	3	3	0.6106 ± 0.0053	0.7705 ± 0.0074	0.6294 ± 0.0076	0.7930 ± 0.0103	0.6905 ± 0.0128	0.8765 ± 0.0164
Dropout (reference = 0.3)	0	3	0.6118 ± 0.0050	0.7721 ± 0.0066	0.6312 ± 0.0071	0.7952 ± 0.0096	0.6929 ± 0.0115	0.8796 ± 0.0154
Dropout (reference = 0.3)	0.5	3	0.6144 ± 0.0048	0.7753 ± 0.0067	0.6347 ± 0.0069	0.7997 ± 0.0093	0.6979 ± 0.0116	0.8859 ± 0.0149

**Hyperparameter reference**	**Setting**	**N**	**6 h MAE**	**6 h RMSE**	**24 h MAE**	**24 h RMSE**	**48 h MAE**	**48 h RMSE**
Learning rate (reference = 8 × 10^{-4})	3 × 10^{-4}	3	0.6131 ± 0.0039	0.7737 ± 0.0051	0.6330 ± 0.0055	0.7975 ± 0.0075	0.6954 ± 0.0089	0.8827 ± 0.0119
Learning rate (reference = 8 × 10^{-4})	2 × 10^{-3}	3	0.6182 ± 0.0058	0.7801 ± 0.0081	0.6401 ± 0.0083	0.8064 ± 0.0112	0.7052 ± 0.0140	0.8952 ± 0.0179
Hyperparameter reference	Setting	N	6 h MAE	6 h RMSE	24 h MAE	24 h RMSE	48 h MAE	48 h RMSE
Adopted reference configuration	64 hidden units; 2 layers; dropout 0.3; learning rate 1 × 10^{-3}	3	0.6472 ± 0.0044	0.8204 ± 0.0061	0.6807 ± 0.0093	0.8596 ± 0.0117	0.7803 ± 0.0135	0.9965 ± 0.0180
Hidden units (reference = 64)	32	3	0.6572 ± 0.0042	0.8330 ± 0.0055	0.6949 ± 0.0084	0.8776 ± 0.0110	0.8009 ± 0.0121	1.0228 ± 0.0161
Hidden units (reference = 64)	128	3	0.6490 ± 0.0056	0.8227 ± 0.0077	0.6833 ± 0.0112	0.8629 ± 0.0148	0.7840 ± 0.0170	1.0013 ± 0.0216
Layers (reference = 2)	1	3	0.6558 ± 0.0041	0.8313 ± 0.0054	0.6930 ± 0.0083	0.8751 ± 0.0109	0.7981 ± 0.0120	1.0192 ± 0.0159
Layers (reference = 2)	3	3	0.6535 ± 0.0059	0.8284 ± 0.0081	0.6898 ± 0.0119	0.8710 ± 0.0156	0.7934 ± 0.0180	1.0132 ± 0.0228
Dropout (reference = 0.3)	0	3	0.6526 ± 0.0054	0.8273 ± 0.0072	0.6885 ± 0.0109	0.8694 ± 0.0144	0.7915 ± 0.0158	1.0108 ± 0.0211
Dropout (reference = 0.3)	0.5	3	0.6549 ± 0.0052	0.8302 ± 0.0072	0.6917 ± 0.0105	0.8735 ± 0.0138	0.7962 ± 0.0159	1.0168 ± 0.0202
Learning rate (reference = 1 × 10^{-3})	3 × 10^{-4}	3	0.6563 ± 0.0043	0.8319 ± 0.0056	0.6936 ± 0.0086	0.8759 ± 0.0114	0.7990 ± 0.0125	1.0204 ± 0.0166
Learning rate (reference = 1 × 10^{-3})	3 × 10^{-3}	3	0.6626 ± 0.0063	0.8399 ± 0.0086	0.7027 ± 0.0126	0.8874 ± 0.0165	0.8121 ± 0.0190	1.0372 ± 0.0242

Hyperparameter reference	Setting	N	6 h MAE	6 h RMSE	24 h MAE	24 h RMSE	48 h MAE	48 h RMSE
Adopted reference configuration	120 boosting rounds; learning rate 0.05; MaxNumSplits 16; MinLeafSize 5	3	0.7066 ± 0.0047	0.8988 ± 0.0065	0.7518 ± 0.0075	0.9501 ± 0.0097	0.8991 ± 0.0115	1.1575 ± 0.0154
Boosting rounds (reference = 120)	80	3	0.7155 ± 0.0051	0.9101 ± 0.0067	0.7647 ± 0.0078	0.9663 ± 0.0104	0.9185 ± 0.0119	1.1825 ± 0.0160
Boosting rounds (reference = 120)	240	3	0.7082 ± 0.0050	0.9019 ± 0.0069	0.7539 ± 0.0076	0.9602 ± 0.0102	0.8972 ± 0.0121	1.1622 ± 0.0156
MaxNumSplits (reference = 16)	8	3	0.7135 ± 0.0046	0.9076 ± 0.0060	0.7618 ± 0.0070	0.9627 ± 0.0094	0.9142 ± 0.0106	1.1769 ± 0.0143
MaxNumSplits (reference = 16)	32	3	0.7111 ± 0.0059	0.9045 ± 0.0081	0.7582 ± 0.0090	0.9582 ± 0.0121	0.9088 ± 0.0144	1.1700 ± 0.0184
Learning rate (reference = 0.05)	0.02	3	0.7190 ± 0.0044	0.9145 ± 0.0058	0.7697 ± 0.0067	0.9727 ± 0.0090	0.9261 ± 0.0102	1.1922 ± 0.0137
Learning rate (reference = 0.05)	0.10	3	0.7125 ± 0.0057	0.9063 ± 0.0079	0.7604 ± 0.0087	0.9609 ± 0.0117	0.9120 ± 0.0139	1.1742 ± 0.0178
MinLeafSize (reference = 5)	2	3	0.7038 ± 0.0061	0.8952 ± 0.0082	0.7482 ± 0.0094	0.9454 ± 0.0121	0.8935 ± 0.0138	1.1505 ± 0.0182
MinLeafSize (reference = 5)	10	3	0.7118 ± 0.0050	0.9054 ± 0.0068	0.7596 ± 0.0082	0.9598 ± 0.0108	0.9109 ± 0.0128	1.1726 ± 0.0169

note: Values are mean ± SD across three fixed-seed validation runs. In each one-factor-at-a-time comparison, only the listed hyperparameter was changed. The adopted reference configuration was retained when its average normalized recursive error was within 1% of the lowest value in the corresponding parameter block, considering run-to-run variability, model complexity, and regularization. The test set was not used for hyperparameter selection.

Supplementary Figure S1. Diagnostic comparison of the linear and Residual-GBRT exogenous-variable generators.

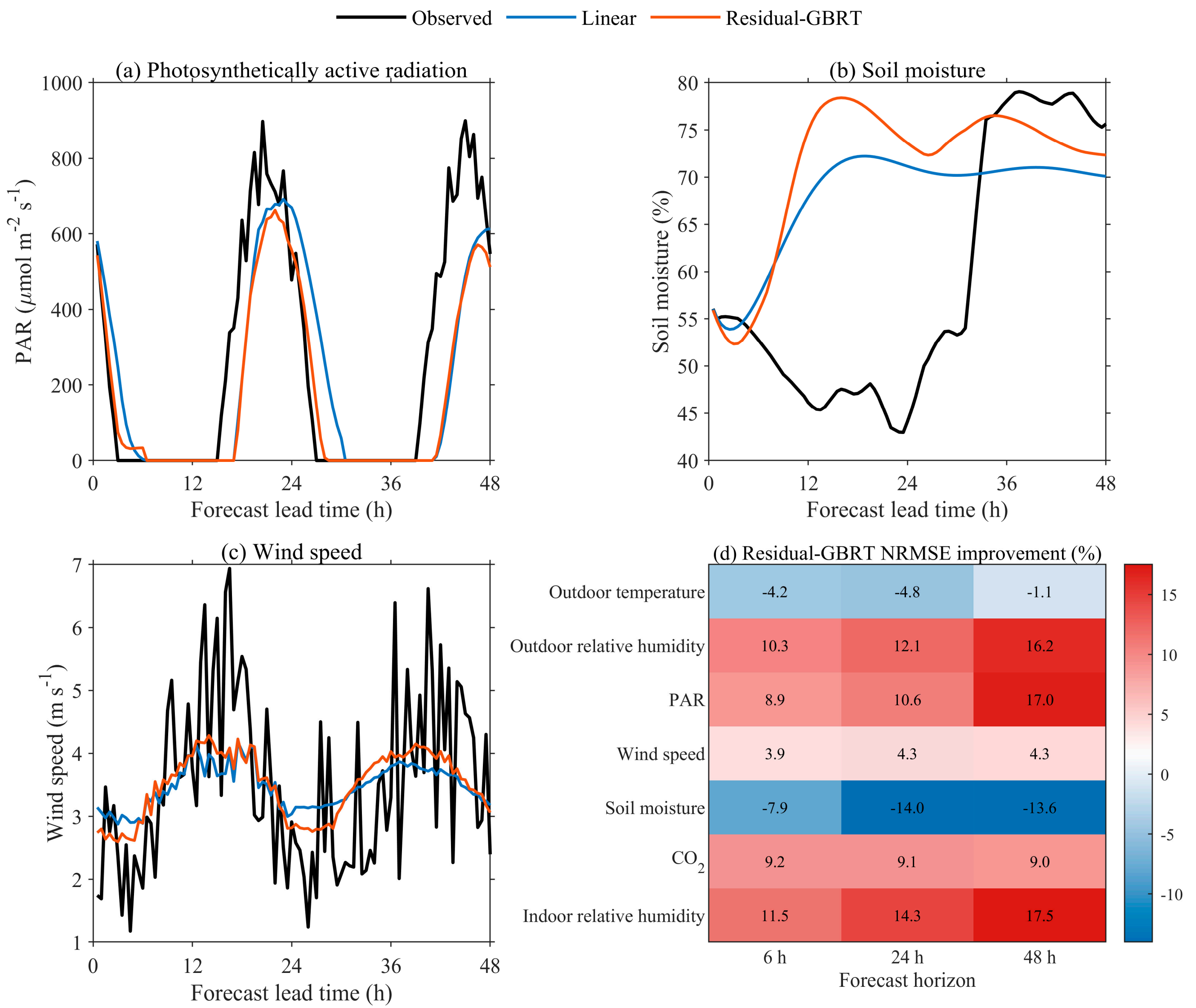

Supplementary Figure S1. Diagnostic comparison of the linear and Residual-GBRT exogenous-variable generators: Representative observed and recursively generated trajectories over a 48 h forecasting window are shown for (**a**) photosynthetically active radiation, (**b**) soil moisture, and (**c**) wind speed. Panel (**d**) presents the percentage change in NRMSE achieved by Residual-GBRT relative to the linear generator under the 6 h, 24 h, and 48 h forecasting horizons. Positive values indicate a reduction in NRMSE, whereas negative values indicate deterioration.
